# Optical biosensors for diagnosis of COVID-19: nanomaterial-enabled particle strategies for post pandemic era

**DOI:** 10.1007/s00604-024-06373-6

**Published:** 2024-05-10

**Authors:** Yusuf Samil Tekin, Seyda Mihriban Kul, Osman Sagdic, Nadnudda Rodthongkum, Brian Geiss, Tugba Ozer

**Affiliations:** 1 Department of Biomedical Engineering, Graduate Education Institute, Malatya Turgut Ozal University, 44210 Battalgazi, Malatya Turkey; 2 Department of Food Engineering, Faculty of Chemical-Metallurgical Engineering, Yildiz Technical University, 34220 Istanbul, Turkey; 3 Metallurgy and Materials Science Research Institute, Chulalongkorn University, Soi Chula 12, Phayathai Road, Bangkok, 10330 Patumwan Thailand; 4 Department of Microbiology, Immunology, and Pathology, Colorado State University, Fort Collins, CO 80523-1019 USA; 5 Department of Bioengineering, Faculty of Chemical-Metallurgical Engineering, Yildiz Technical University, 34220 Istanbul, Turkey; 6 Health Biotechnology Joint Research and Application Center of Excellence, Esenler 34220 Istanbul, Turkey

**Keywords:** COVID-19, Virus, Optical detection, SARS-CoV-2, Biomarkers, Machine learning

## Abstract

The COVID-19 pandemic underlines the need for effective strategies for controlling virus spread and ensuring sensitive detection of SARS-CoV-2. This review presents the potential of nanomaterial-enabled optical biosensors for rapid and low-cost detection of SARS-CoV-2 biomarkers, demonstrating a comprehensive analysis including colorimetric, fluorescence, surface-enhanced Raman scattering, and surface plasmon resonance detection methods. Nanomaterials including metal-based nanomaterials, metal–organic frame–based nanoparticles, nanorods, nanoporous materials, nanoshell materials, and magnetic nanoparticles employed in the production of optical biosensors are presented in detail. This review also discusses the detection principles, fabrication methods, nanomaterial synthesis, and their applications for the detection of SARS-CoV-2 in four categories: antibody-based, antigen-based, nucleic acid–based, and aptamer-based biosensors. This critical review includes reports published in the literature between the years 2021 and 2024. In addition, the review offers critical insights into optical nanobiosensors for the diagnosis of COVID-19. The integration of artificial intelligence and machine learning technologies with optical nanomaterial-enabled biosensors is proposed to improve the efficiency of optical diagnostic systems for future pandemic scenarios.

**Graphical Abstract:**

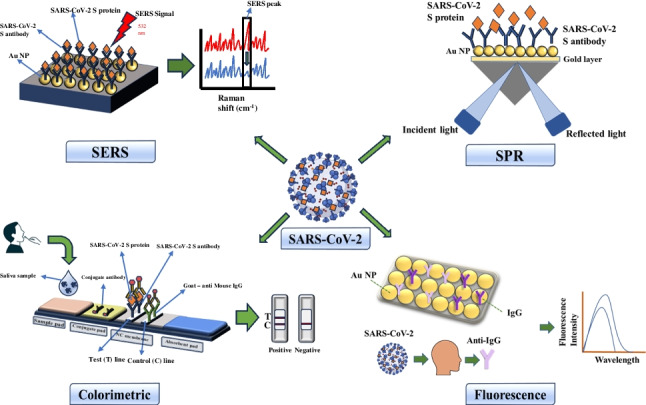

## Introduction

COVID-19 has become a global pandemic affecting and challenging the lives of millions of people and communities all over the world, which is highly infectious airborne disease that emerged at the end of 2019 in Wuhan, China, resulting in more than 800 million cases so far [1, 2]. SARS-CoV-2 virus causes COVID-19 with symptoms including cough, fever, headache, and loss of taste and smell [3]. The SARS-CoV-2 virus is transmitted by droplets spread from person to person by sneezing and coughing [4, 5]. Due to the high number of asymptomatic patients and the fact that they are as contagious as symptomatic patients, early diagnosis of COVID-19 becomes important [2, 6]. Most vaccination methods for SARS-CoV-2 target the spike protein, as generation of strong neutralizing antibodies against spike is correlated with blockade of virus particles being able to enter the host cells and disruption of the viral infection cycle [7, 8].

SARS-CoV-2, the causative agent of COVID-19, is a large RNA virus belonging to the beta coronavirus family that possesses a 5′-capped and 3′ polyadenylated single-stranded RNA genome of approximately 30 kilobases in size [9]. The viral genome encodes a total of 29 proteins, including 16 nonstructural proteins, 4 structural proteins, and 9 accessory proteins. The structural proteins are critical for the virus’s physical makeup, while the nonstructural and accessory proteins play various roles in the virus’s replication and its interaction with the host’s immune system. A description of the organization of the viral genome and the roles of the different viral proteins in various aspects of the viral lifecycle can be found elsewhere [9].

The SARS-CoV-2 virus particle is membranous, approximately 120 nm in diameter, and studded with the spike (S), membrane (M), and envelope (E) proteins which serve different roles during the viral lifecycle [10, 11]. The spike glycoprotein is the major immunological antigen for SARS-CoV-2 (along with nucleocapsid), and the major role of the mature spike trimer is to associate with the angiotensin-converting enzyme 2 (ACE2) proteins on host cells to initiate entry of the virus particle into target cells [12]. The SARS-CoV-2 RNA genome is coated by nucleocapsid protein in virus particles and during replication [13]. The nucleocapsid proteins are critical to the viral lifecycle, as they condense the large viral genome (similar to chromatin in host cells), help direct the viral genome for packaging into the viral particle, and help protect the viral genome from antiviral processes while in the cell.

The nucleocapsid protein is the main protein diagnostic marker for active SARS-CoV-2 infection, as nucleocapsid proteins are only present during active infection. The high concentration of the nucleocapsid protein present in viral particles allows detection of even relatively low concentrations of particles in infection, making nucleocapsid the preferred antigen for lateral flow-based antigen tests. Spike is present at a lower concentration on viral particles than nucleocapsid, making it less useful for antigen testing. The detection of nucleocapsid from virus particles requires disruption of the particles in a manner that maintains the protein’s native structure that affinity molecules can bind to, which is commonly performed in antigen tests using various surfactants to dissolve the virus particle’s lipid membranes and uncover the nucleocapsid.

For the detection of antibody responses against vaccinations or primary infections, diagnostic tests use the spike protein as an antigen. For clinical PCR-based diagnostic testing for sensitively detecting viral genomes, reverse transcription of RNA in samples to DNA and then amplification with oligonucleotide primers targeting either (or both) spike and nucleocapsid gene sequences is commonly performed due to the relatively high levels of spike and nucleocapsid mRNAs generated during infection [9]. Conventional techniques including real-time reverse transcription polymerase chain reaction (RT-PCR) [14, 15] and enzyme-linked immunosorbent assay (ELISA) usually employ respiratory specimens [16]. The respiratory specimens collected from the upper tract include viral infection in 7 days with high viral loads, whereas the lower respiratory tract contains less viral loads compared to nasal samples [17]. In addition, it might not be possible to detect viral RNA in nasal or throat swabs in 2 weeks after symptoms are seen. Among conventional methods, RT-PCR is considered the gold standard method for detecting SARS-CoV-2 [18, 19]. However, traditional methods are not ideal in that they have long response time, require time-consuming sample preparation steps, are laboratory based, and require expensive equipment [20, 21]. Although immunoassay-based technologies have been mostly used with blood serum as the clinical sample, saliva and stool specimens can be employed for respiratory viral samples, requiring fewer sampling procedures. Moreover, sensitivity and minimized false negative test results could be achieved by testing samples from different sites.

A variety of COVID-19 diagnosis methods have been developed to decrease mortality rates and improve survival rates. Among all, biosensors are considered promising early detection techniques for screening purposes [22]. Biosensors are superior analytical devices compared to traditional detection methods owing to their practicability use, such as affordability, fast response, high sensitivity, and portability [23]. After the interaction of the receptor with the analyte, signal generation occurs with the transducer and the signal is transferred to the sensing system for conversion into analyzable information [24]. According to the type of signal output, the biosensors are divided into electrochemical, optical, calorimetric, piezoelectric, and thermoelectric [25].

Among these techniques, optical biosensors are highly preferred due to their affordable cost, simple operation, and high technology. In addition, optical sensors can be used as point-of-care (POC) diagnostic tools without the need for professionals [26]. In optical sensors, the transducer is added to the light source and changes such as absorption, refraction, and reflection occur in the light in response. According to these changes, the input analyte can be analyzed [25]. Surface plasmon resonance (SPR) [27, 28], fluorescence [29, 30], surface-enhanced Raman scattering (SERS) [31, 32], and colorimetric detection are methods used for optical biosensors [33]. Surface plasmon occurs when photons in the incident wave encounter electrons on the surface of the metal and are analyzed due to the shift in the refractive index caused by this encounter [34]. Fluorescence, which is a result of analyte absorption when it is exposed to an external light source, is typically analyzed using fluorophore molecules, wavelength filters, and detectors [25]. SERS biosensors generate Raman signals in analytes using the superior sensing capabilities of SERS-active substrates via Raman spectroscopy [32, 35]. The nanotechnology-enabled Raman spectroscopy is highly successful to assess SARS-CoV-2 with high specificity. Magnetic plasmonic nanoparticles are employed to the control SERS substrate, whereas a noble metal shell offers efficient plasmonic activity. The working principle of colorimetric biosensors is based on color change observed with the naked eye. Among optical biosensors, colorimetric biosensors are mostly used as POCs due to their features such as direct reading, portability, and low cost [26, 35].

There are few reports on optical biosensors for the diagnosis of COVID-19 [26, 36–38]. However, these reports either present the optical biosensors without nanomaterial modifications or do not cover recent studies published within the last 2 years. While some of these reports lack categorizing the presented works based on the probe type, some of them are focused on solely portable biosensors for the detection of SARS-CoV-2. Moreover, paper-based biosensors and lateral flow assays were mostly emphasized in those reports. In this review, we present an overview of the most developed nanomaterial-enabled optical biosensors including colorimetric mode, SERS mode, SPR mode, and fluorescence mode for the detection of SARS-CoV-2. Also, the review discusses potentials and future trends and directions for the application of these biosensors.

### Antibody-based biosensors

Affinity molecules used in sensors that detect viral antigens can be broadly categorized into two main categories: antibodies and aptamers. Antibody is a natural affinity molecule that is generated in mammalian organisms as a response to infection, and whose maturation process results in high-affinity biomolecules that can selectively bind to specific molecular features (known as epitopes) on chemical or protein targets (e.g., antigens). Antibodies for diagnostic assays are traditionally generated by injecting the antigen of interest into a mammalian animal (rabbits, mice, goats, donkeys, etc.), and polyclonal serum can be collected from the animal at some time after immunization. These polyclonal sera generally contain multiple high-affinity antibodies towards multiple epitopes on the antigen of interest and are used commonly in research applications due to activity and relatively low cost, but the lack of well-defined epitopes and lot-to-lot variability make them inappropriate for clinical diagnostic test usage. Monoclonal antibodies are more commonly used for clinical diagnostic tests. Monoclonal antibodies are generated from B-cells isolated from the immunized animals that are subsequently immortalized and selected for their abilities to produce single high-affinity antibodies towards well-defined epitopes. A number of monoclonal antibodies have been developed that specifically bind SARS-CoV-2 antigens [39–42]. Monoclonal antibodies, which can be generated in large qualified batches via contract research organizations, are commonly used in clinical diagnostic assays including enzyme-linked immunosorbent assays (ELISA), plaque reduction neutralization assays (PRNT), and lateral flow assays (LFA). There are various antibody-based nanomaterial-modified biosensors in the literature. The works presented in this section are summarized in Table 1.

**Table 1:** Antibody-based biosensors

Nanomaterial	Optical method	Linear range	LOD	Real sample	Reference
Au NPs	SERS	100 μg/mL–0.01 ng/mL	0.046 ng/mL	-	[43]
Ag NPs	SERS	1000 ng/mL–100 pg/mL	400 pg/mL	Saliva	[44]
AgMENs	SERS	1.0 fg/mL–1.0 mg/mL	1.0 fg/mL	-	[45]
FAPbI_3_ PNC	SPR	-	10^−6^ RIU	-	[46]
Au@Ag@Au	SPR	0.1–1000 ng/mL	0.083 ng/mL	Spiked saliva sample	[47]
AuNPs	SERS	1 fg/mL–1 ng/mL	6.07 fg/mL	Untreated saliva	[48]
AuNPs	Colorimetric	25–200 μg/mL	13.75 μg/mL	Human saliva	[49]
Cellulose paper	Colorimetric	100–10 ng/mL	31.6 ng/mL	Human serum	[50]
Au@PtNPs	Colorimetric	0.1–1000 pg/mL	0.1 pg/mL	Throat swab	[51]
UCNPs and AuNRs	Luminescence	2–32 fg/mL	1.06 fg/mL	Saliva sample	[52]
POF and GOF	P-FAB	-	2.5 ng/mL	-	[53]
NPG	LSPR	-	319 copies/mL	Artificial saliva	[54]
NHGNPs	Nanoplasmic	-	0.2 pM	Serum samples without SARS-CoV-2 infection	[55]
Au NPs	Colorimetric	-	5 × 10^4^ copies/mL	Nasopharyngal swab	[56]
MnO_2_QDs@Lip	Colorimetric	0.1 pg/mL–100 ng/mL	65 fg/mL	Human sample	[57]
Au@CeO_2_@Pt	Colorimetric–fluorescence	0.1–200 ng/mL 0.1–100 ng/mL	0.062 ng/mL 0.036 ng/mL	-	[58]
Si@Au@PEI NPs	Colorimetric–fluorescence	0.1–0.005 ng/mL	0.5 ng/mL 0.025 ng/mL	Pharyngeal swab	[59]
MagAu_shell_ NP	Colorimetric – photothermal	-	1 ng/mL 43.64 pg/mL	Saliva	[60]
AgMBA@Au NPs	SERS	-	10^−6^ mg/mL	Serum	[61]
Au NPs	Colorimetric–SERS	-	500 PFU/mL 5.2 PFU/mL	Human sample	[62]
Au-Fe_3_O_4_ Nps	Colorimetric–Photothermal	10^2^–10^6^ pg/mL	10^4^ copies/mL 1.22 pg/mL	Pseudovirus	[63]
Ti3C2-QD nanoflakes	Colorimetric–Fluorescence	-	1 ng/mL 6.2 pg/mL	Throat swab	[64]
SiO_2_@Au/QD	Colorimetric and fluorescence	-	1 ng/mL 33 pg/mL	Spiked saliva samples	[65]
AuNPs	Nanoplasmic	-	0.01 ng/mL	-	[67]
Au@PtNPs	NLICS	0.05–1.6 ng/mL	0.026 ng/mL	Blood sample	[68]
SiO_2_@Ag NPs	SERS	10–0.001 ng/mL	1 pg/mL	-	[69]
AuTNP	LSPR	-	63.6 aM	Plasma samples	[70]
Au@PtNPS	Colorimetric	10–100 ng/mL	11 ng/mL	-	[71]
OPOCT	Fluorescence	12.5–1000 ng/mL	12.5 ng/mL	-	[72]
CoFe/NC@Pt	Colorimetric–fluorescence	0.05–100 ng/mL	0.022 ng/mL 0.018 ng/mL	Human serum	[73]
Au/FBG	SPR	-	1.6 × 10^8^ copies/mL	Saliva samples	[74]
COP	IPCF	1–100 pg/mL	429 fg/mL	Artificial saliva	[75]

Bistaffa et al. designed a SERS-based sensor for the detection of SARS-CoV-2 spike protein [43]. The sensor was based on a design in which methylene blue as a Raman reporter was coated with a silica shell and adsorbed on gold nanoparticles (AuNPs). AuNPs were first synthesized and then attached to methylene blue via covalent bonding through N-CH_3_ groups of methylene blue. (3-Aminopropyl)triethoxysilane (APTMS) was added to AuNPs, followed by the addition of sodium silicate solution to generate silica shells. The resulting SERS probes were mixed with (3-triethoxysilyl) propylsuccinic anhydride (TEPSA) to immobilize antibodies. The biosensor had a linear range between 100 μg/mL and 0.01 ng/mL with an LOD of 0.046 ng/mL. Another SERS biosensor was developed by Kaladharan et al. [44]. In the preparation of SERS substrates, nanostructures were first formed on digital versatile discs (DVD discs) via the physical vapor deposition method. The DVD substrate was coated with titanium and silver NPs. In addition, the polyethylene glycol (PEG) binder was incubated on a modified SERS substrate to immobilize the SARS-CoV-2 antibody. The LODs were found to be 50 pg/mL and 400 pg/mL for spike and virus-like particle (VLP) proteins in saliva, in the linear range of 1000 ng/mL–100 pg/mL, respectively, in 20 min with the use of a portable Raman spectrometer. Similarly, Yeh et al. developed a silver microplasma-engineered nanorods (AgMENs)–modified Raman scattering SERS biosensor for the detection of SARS-CoV-2 spike protein and nucleocapsid proteins [45]. Cellulose papers were used for the deposition of nanoparticles. Then, the SERS substrate was incubated with anti-S IgG SARS-CoV-2 antibodies, and then non-specific regions were blocked with BSA. The linear range of the SERS biosensor was determined to be 1.0 fg/mL to 1.0 mg/mL with an LOD of 1.0 fg/mL. In addition, the biosensor had stability for 20 days.

**Fig. 1:**
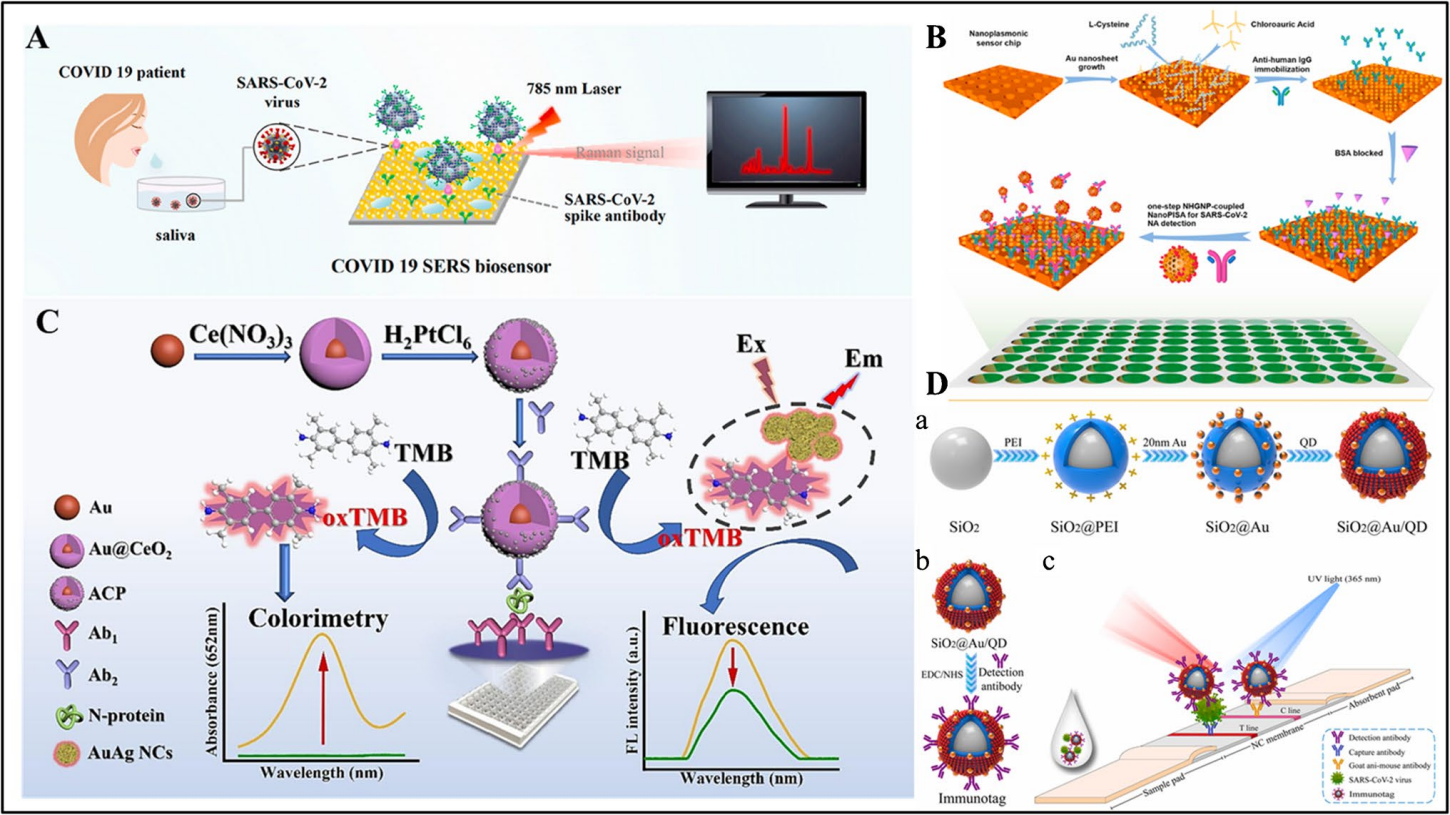
**A** Schematic illustration of the SERS-based immunoassay in untreated saliva. Reproduced with permission from Ref. [48]. **B** One-step rapid quantification of total SARS-CoV-2 NAs with the novel nanoparticle-coupled biosensor platform. Reproduced with permission from Ref. [55]. **C** Illustration of the assay mechanism of dual-mode immunoassay. Reproduced with permission from Ref. [58]. **D** (a) Sequential process for fabricating dual-functional SiO_2_@Au/QD fluorescent labels. (b) Preparation of S protein-conjugated SiO_2_@Au/QD labels. (c) Schematic of a dual-functional LFIA biosensor. Reproduced with permission from Ref. [66]

Chen et al. developed a wavelength-based SPR biosensor [46]. Air-resistant NIR-emitting perovskite nanocomposites (PNC) were used as the light source of the biosensor. For the synthesis of NIR-emitting formamidinium lead tri-iodide (FAPbI_3_) PNC, formamidine acetate, and octanoic acid were mixed in the presence of lead (II) iodide at 80 °C. Then, (3-aminopropyl)triethoxysilane (APTES) was added to the mixture while cooling at 25 °C. The final composite was mixed with UV adhesive and coated on Indium Gallium Nitride (InGaN)–based blue LEDs to obtain NIR-quantum-dot light-emitting diode (QLED). QLEDs were used as quantum dot light-emitting diodes, converting light from the NIR. The sensor surface was coated with Au monolayers, and then the anti-SARS-CoV-2 spike polyclonal capture antibody was covalently immobilized with a commercial amine coupling kit. The refractive index (RIU) sensitivity of the SPR sensor was 10^−6^ RIU in less than 15 min. In another report, Fan et al. developed an SPR biosensor for SARS-CoV-2 N protein detection in artificial saliva [47]. At first, AuNPs were synthesized. Then, a mixture of AgNO_3_ and ascorbic acid was added to the AuNP colloids to obtain Au@Ag@Au core–shell nanospheres. An Au chip was activated using β-mercaptoethylamine (MEA) and immersed in graphene oxide (GO) solution to replace amino groups while carboxyl functional groups were activated using EDC/NHS to immobilize Ab1. A sandwich immunoassay based on Au@Ag@Au NPs/Ab2/SARS-CoV-2 N protein/Ab1 was obtained by injection of Au@Ag@Au/Ab2/ SARS-CoV-2 conjugate into the chip. The biosensor showed a linear range of 0.1–1000 ng/mL with an LOD of 0.083 ng/mL. An average recovery of 109.21% was obtained for SARS-CoV-2 N protein detection in spiked saliva samples. In another report, Zhang et al. presented a SERS-based biosensor for the detection of SARS-CoV-2 spike protein in saliva (Fig. 1A) [48]. At first, AgNO_3_, ascorbic acid (AA), and trisodium citrate were mixed to prepare silver nanoparticles (AgNPs). The AgNPs were then incubated with 4-mercaptobenzoic acid (4MBA) to immobilize the SARS-CoV-2 spike antibody, followed by blocking non-specific sites with BSA. AuNPs were then synthesized and placed on hydrophilic silicon wafers to immobilize the SARS-CoV-2 spike antibody. Finally, SERS peak intensity was observed at 1077 cm^−1^ in the linear range between 1 fg/mL and 1 ng/mL. The LODs were 0.77 fg/mL and 6.07 fg/mL for the target antigen in PBS and untreated saliva, respectively. Furthermore, the serum and blood samples were tested and LODs were found to be 7.60 fg/mL and 0.10 pg/mL, respectively.

Punnoy et al. reported a lab-on-paper screening-based colorimetric biosensor to detect the SARS-CoV-2 Omicron BA.2 variant in human saliva [49]. The lab-on-paper platform was fabricated using wax printing on filter paper. Colorimetric reactions were performed in a 3-mm diameter detection zone on a paper substrate. For colorimetric detection, AuNP-Ab bioconjugate was drop-cast on the detection area. After drying, it was tested by incubating with SARS-CoV-2 antigen. The change in color signal according to concentration was analyzed with ImageJ software. The biosensor demonstrated a linear range of 25–200 μg/mL with an LOD of 13.75 μg/mL in human saliva for SARS-CoV-2 detection. The biosensor has the advantage that it is possible to visualize the sensor response without the need for an instrument.

In another report, Correira et al. developed a cellulose-based colorimetric sensor for detection of the SARS-CoV-2 [50]. Cellulose papers with a diameter of 0.6 cm were cut and oxidized with sodium methaperiodate (NaIO_4_) for 1 day in a light-free environment to form aldehyde groups on the sensor surface. S protein was then covalently bound to the functionalized surface via amine groups of the protein. Finally, human anti-SARS-CoV-2 antibodies were incubated on the biosensor surface and antibody quantification was performed in the presence of Ponceasu S solution, giving off a red color. The biosensor had a linear response between 100 and 10 ng/mL with an LOD value of 31.6 ng/mL in diluted human serum samples in 30 min. Another colorimetric biosensor was developed by Wu et al. [51]. The colorimetric microfluidic chip was based on platinum-decorated gold nanoparticles (Au@PtNP) for the detection of SARS-CoV-2 N protein. Au@PtNPs were synthesized and functionalized with dithiothreitol (DTT) to form antibody conjugates. Then, a mixture of casein and glutathione solution was used as a blocking agent. The magnetic beads were coated with streptavidin and biotinylated antibodies were then incubated. The microfluidic chip consists of two polydimethylsiloxane (PDMS) layers, a polymethylmethacrylate (PMMA) layer, and a polyethylene terephthalate (PET) film layer. The prepared Au@PtNP-antibody conjugates were then incubated. 3,3′,5,5′-tetramethylbenzidine (TMB) substrate solution was added to the third inlet port. Once the TMB substrate reacted with the captured structures, the LOD value of the chip in the linear range of 0.1–1000 pg/mL was determined to be 0.1 pg/mL. The chip was found to be selective against SARS-CoV-2 N protein (omicron) and SARS-CoV N protein and gave positive results.

Li et al. reported an upconversion nanoparticle (UCNP) luminescence biosensor for the detection of SARS-CoV-2 spike protein [52]. Gold nanorods (AuNRs) were synthesized, followed by the addition of AgNO_3_. After thiolation of anti-SARS-COV19 IgM, AuNRs were conjugated with the antibodies. Swaps containing SARS-CoV-2 were immobilized on bare UCNPs (B-UCNP) due to the electrostatic attraction. The biosensor demonstrated an LOD of 1.06 fg/mL SARS-CoV-2 spike protein in the linear detection range from 2 to 32 fg/mL in 5 min. The recovery rate was found to be 80–120% in saliva samples.

Divagar et al. reported a plasmonic fiberoptic absorbance biosensor (P-FAB) to detect SARS-CoV-2 nucleocapsid protein [53]. A sandwich immunoassay using AuNP labels was applied on a U-bent multimode fiber optic probe surface. The effect of optical fiber material on the physisorption of antibodies was investigated by fabricating a plastic optical fiber (POF) and a glass optical fiber (GOF). Both POF and GOF probes were covalently immobilized with anti-SARS CoV-2 N-protein IgG1 overnight. After a sandwich structure AuNP-IgG2-SARS CoV-2 N-protein-IgG1 complex formed on the GOF probe surface, AuNP labels absorbed light passing through the U-bent region, resulting in increased concentration of the analytes. The LODs were 17.78 ng/mL and 5.62 ng/mL for POF and GOF probes, respectively. Therefore, GOF sensor probes were used to detect SARS-CoV-2-N-protein detection. The biosensor was tested for SARS-CoV-2 nucleocapsid protein detection at 530 nm, and the LOD was obtained as 2.5 ng/mL in PBS within 10 min. The biosensor has the advantage that it is possible to be used at the POC due to the presence of portable a green light-emitting diode (LED), a photodetector, and bare fiber adaptors.

Zheng et al. reported a label-free vertical microcavity and localized surface plasmon resonance (LSPR) biosensor for the detection of SARS-CoV-2 in artificial saliva [54]. The microcavity LSPR biosensor was fabricated by anodization, oxidation, deposition, and biofunctionalization steps. The substrate of the nanoporous gold (NPG) sensor was formed by depositing SiO_2_ vertical microcavity, adhesive layer, barrier layer, and Au–Ag alloy thin film formed after anodization and oxidization on silicon wafers, respectively. Afterwards, antibody immobilization was performed by preparing the SARS-CoV-2 antibody. The SARS-CoV-2 pseudovirus was spiked in artificial saliva and tested with the developed biosensor. The sensing system of the sensor consists of a real-time spectrum monitoring interface, light source, spectrometer, Y-shaped optical fiber, robotic arm system, and sensing software. The biosensor showed an LOD of 319 copies/mL in 30 min. The vertical microcavity-LSPR sensor has a short turnaround with a full-automated system. In another report, Huang et al. developed a nanostructure-coupled biosensor platform for one-step high-throughput measurement of serum neutralizing antibody after COVID-19 vaccination (Fig. 1B) [55]. The nanoplasmonic sensor chip was fabricated by a copy molding technique. It was then placed on polyethylene terephthalate, and titanium and gold were deposited. Finally, the chip was cut and glued onto a 96-well plate to obtain a chip. Colloidal gold nanoparticles (GNP) were formed from HAuCl_4_ and citrate reduction and then incubated with S-RBD. SARS-CoV-2 NA solution was added to the anti-human IgG functionalized sensor chip plate. This biosensor demonstrated an LOD of 0.2 pM in 15 min. The level of SARS-CoV-2 NA was determined in serum samples before and after COVID-19 vaccination in patients without SARS-CoV-2 infection.

Lee et al. reported a colorimetric lateral flow immunoassay based on oriented antibody immobilization for sensitive detection of SARS-CoV-2 [56]. Firstly, AuNPs were conjugated with SARS-CoV-2 spike antibody. Cellulose-binding protein B and C (CBP31-BC) domains were incubated with the antibody to form a capture antibody. The capture antibody and CBP31-BC were then placed on the test line and control line respectively. Diluted samples were transferred to the sample pad and let flow on the conjugate pad to allow the formation of antigen–antibody complex. The LOD was determined to be 5 × 10^4^ copies/mL in 15 min. Nasopharyngeal swab samples from COVID-19 patients and healthy individuals were used to validate the lateral flow assays (LFA).

Chu et al. developed a colorimetric biosensor for the detection of SARS-CoV-2 nucleocapsid antigen (CoVN1) using quantum dot–sized liposome-encapsulated MnO_2_ nanozymes (MnO_2_QDs@Lip) as a signaling mediator [57]. At first, MnO_2_QDs@Lip were synthesized. For SARS-CoV-2 detection, MBs-Ab_1_ and CoVN1 in PBS were first mixed and MBs-Ab_1_-CoVN1 was collected with a magnet. Then, MnO_2_QDs@Lip-Ab_2_ was added to this mixture. Finally, the resulting MBs-Ab_1_-Ag-Ab_2_-MnO_2_QDs@Lip (MBs-Ag-MnO_2_Qds@Lip) was collected with a magnet. The antigen captured by these antibodies converts colorless TMB to blue color, providing colorimetric detection in 20 min measured at 650 nm using a plate reader. The LOD of the biosensor in the range of 0.1 pg/mL to 100 ng/mL was 65 fg/mL. The biosensor was stable for 2 months when it was kept at − 20 °C.

Cao et al. developed a nanozyme-linked immunosorbent assay based on Au@CeO_2_@Pt nanozymes capable of both colorimetric and fluorescence detection of the SARS-CoV-2 N protein (Fig. 1C) [58]. Au@CeO_2_ NPs were synthesized. To fabricate the multifunctional biosensor, SARS-CoV-2 nucleocapsid protein antibody 2D3 (Ab_1_) was first added to the 96-well plate and incubated. Then, Au@CeO_2_@Pt-SARS-CoV-2 nucleocapsid protein antibody 3F2 (Ab_2_) conjugates were added to the wells to generate a sandwich assay. Finally, the peroxidase-like activity of Au@CeO_2_@Pt oxidizes TMB, resulting in colorimetric detection. The LOD value of the colorimetric analysis in the linear range of 0.1–200 ng/mL was determined as 0.062 ng/mL, whereas the LOD of the fluorescence analysis in the linear range of 0.1–100 ng/mL was 0.036 ng/mL in simulated urine and saliva. However, the biosensor should be tested with real samples as well. Another dual-mode biosensor combined with colorimetric and fluorescence detection was reported by Yang et al. [59]. A dual signal nanocomposite (SADQD) was obtained by depositing AuNPs and two layers of quantum dots on SiO_2_. The immunochromatographic assay (ICA) strip was prepared using a sample pad, conjugate pad, nitrocellulose (NC) membrane, and absorbent pad. The LODs of the S1 protein were obtained as 0.5 ng/mL and 0.025 ng/mL for colorimetrically and fluorescently, respectively, whereas they were 0.1 ng/mL and 0.005 ng/mL for the N protein. The developed sensor was tested by adding S1 and N proteins to healthy human pharyngeal swabs, and recoveries were obtained as 94.8–101.7% and 92.6–106%, respectively.

Li et al. developed multimodal lateral flow immunoassay (LFIA) tags to detect SARS-CoV-2 nucleocapsid protein in both a colorimetric and photothermal manner [60]. At first, Fe_3_O_4_ nanoclusters and Au nanoshells were synthesized. N protein antibody was added to the final nanocomposite and incubated with BSA. Then, MagAu_shell_ NPs were added to the sample and incubated while detecting N protein on the strip. A test strip was placed in the solution in a centrifuge tube. In the test strip, the samples moving from the sample pad to the absorbent pad reacted with N protein antibodies on the T line in 10 min if N protein was present. While the T line on the test strip was detected qualitatively by the naked eye with an LOD of 1 ng/mL, it was observed quantitatively due to the temperature change under 808-nm laser irradiation with an LOD of 43.64 pg/mL. The developed LFIA strip is applied to artificial saliva and real samples with recovery rates between 93 and 115%.

Liang et al. developed a LFA for both colorimetric and SERS detection of SARS-CoV-2 IgG [61]. AgMBA@Au were formed with the use of polyvinylpyrrolidone (PVP) solution, I-ascorbic acid (I-AA), sodium hydroxide, sodium sulfite, and AgNP solution. The test strip was immersed in the solution of SARS-CoV-2 IgG with AgMBA@AuNPs. While qualitative detection was performed by the line on the T line on the test strip in 15 min, quantitative determination was obtained with a portable Raman spectrometer. In serum samples, the T line was visible at a concentration of 10^−6^ mg/mL. Similarly, Lu et al. developed a SERS-LFA sensor for the simultaneous detection of SARS-CoV-2 N protein and influenza A virus on a single strip [62]. The SERS-LFA strip consists of two test lines and one control line. To prepare SERS nanotags, positively charged malachite green isocyanate (MGITC) was adsorbed on surface-negatively charged AuNPs. SARS-CoV-2 N protein antibody was applied to the first test strip, whereas influenza A N protein antibody was added to the second test strip. The colorimetric detection was obtained up to 500 PFU/mL and 1008 HAU/mL for SARS-CoV-2 and influenza A, respectively. The LOD of the SERS-LFA strip for SARS-CoV-2 was 5.2 PFU/mL and for influenza A virus was 23 HAU/mL.

Guo et al. developed an LFIA test strip modified with Au-Fe_3_O_4_ nanoparticles that enables both colorimetric and photothermal detection of SARS-CoV-2 spike protein [63]. To immobilize the S protein antibody, thiol-poly(ethylene glycol)-carboxyl (HS-PEG-COOH) was added to the Au-Fe_3_O_4_ solution. The S protein antibody was sprayed on the T line of the LFIA strip, whereas the goat anti-mouse antibody was sprayed on the C line. To detect SARS-CoV-2 S protein, Au-Fe_3_O_4_ NPs with S protein antibody captured S protein and gave color to the T line. The LFIA had an LOD value of 1.22 pg/mL in the linear range of 10^2^–10^6^ pg/mL, whereas the LOD was 10^4^ copies/mL for colorimetric detection of SARS-CoV-2 pseudovirus. The LFIA had stability for 8 weeks.

Liu et al. developed an ICA sensor that can simultaneously detect both SARS-CoV-2 N protein and influenza A H1N1 virus colorimetrically and fluorescently [64]. Ti_3_C_2_-QD nanoflakes were prepared in the presence of Ti_3_C_2_ MXene solution and PEI due to the attraction of positive and negative charges. The T1, T2, and C lines on the NC membrane were sprayed with FluA antibody, SARS-CoV-2 N antibody, and goat-antifare IgG, respectively. While the coloration of C and T1 lines indicates the presence of H1N1, the coloration of the C and T2 lines indicates the presence of N protein. The sensor had an LOD of 1 ng/mL for colorimetric detection of the H1N1 virus and the SARS-CoV-2 N protein, whereas it had an LOD of 2.4 pg/mL and 6.2 pg/mL fluorescence detection of H1N1 virus and the SARS-CoV-2 N protein, respectively. Throat swabs infected with H1N1 and SARS-CoV-2 viruses were analyzed and recoveries were between 84.70 and 114%.

In a similar report, Han et al. reported a colorimetric and fluorescence dual-function LFIA to detect SARS-CoV-2 S1 protein (Fig. 1D) [65]. To generate strong fluorescence and colorimetric signals, AuNPs and quantum dots (QDs) were mixed on a SiO_2_ core. To prepare the SiO_2_@Au/QDs-based LFIA biosensor, the detection line of LFIA was sprayed with a capture antibody, whereas the control line was sprayed with a goat anti-mouse IgG antibody. SiO_2_@Au/QD-S_1_ protein was captured with an antibody, resulting in colorimetric and fluorescent bands in the test line in 30 min. The LOD was obtained as 7.06 × 10^5^ copies /mL with the SiO_2_ @Au/QD-LFIA colorimetric function, whereas the LOD was found to be 1.02 × 10^4^ copies/mL for fluorescence detection. Colorimetric and fluorescence detection limits were determined as 1 ng/mL and 33 pg/mL, respectively, with recovery of 95.71% in spiked saliva samples in 30 min. The biosensor has the advantage that visual and quantitative detection of the virus were both possible to detect SARS-CoV-2 infection at an early stage.

**Fig. 2:**
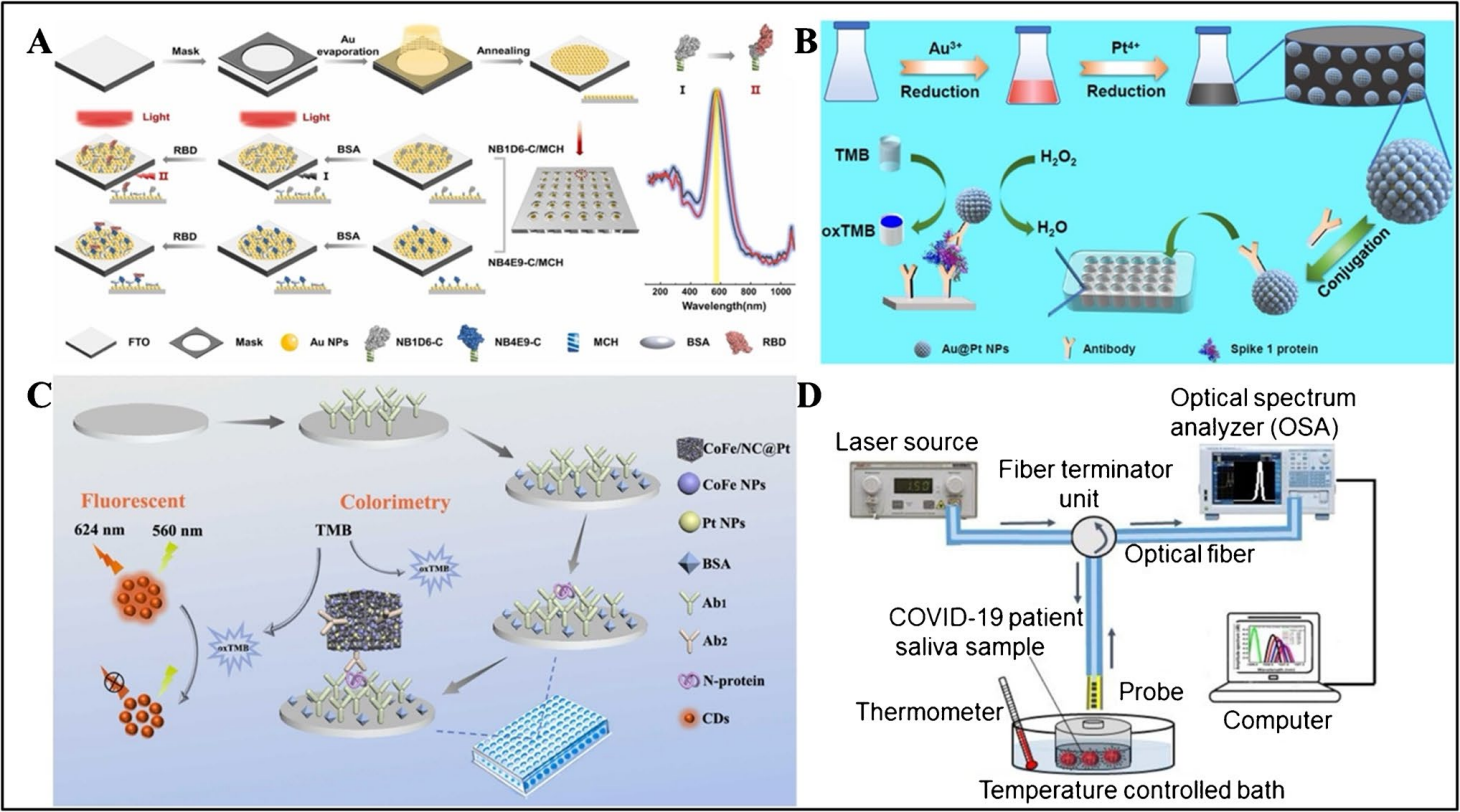
**A** Schematic illustration of nanoplasmonic biosensor for quantification of SARS-CoV-2 spike RBD. Reproduced with permission from Ref. [67]. **B** Illustration of colorimetric detection of spike 1 protein of SARS-CoV-2 based on peroxidase catalysis of Au@PtNPs. Reproduced with permission from Ref. [71]. **C** Construction of colorimetric and fluorescent immunosensor based on CoFe/NC@Pt. Reproduced with permission from Ref. [73]. **D** Schematic diagram of the experimental setup. Reproduced with permission from Ref. [74]

In another report, Ma et al. reported a LSPR-based plasmic biosensor for the detection of SARS-CoV-2 spike receptor-binding domain (RBD) (Fig. 2A) [67]. Firstly, AuNPs were prepared and placed on fluorine-doped tin oxide (FTO) substrates. In the next step, AuNP-coated substrates were placed in a homemade 36-well plate containing antibodies and analyzed by collecting LSPR spectra. The serum samples were added to the plate containing SARS-CoV-2 spike RBD and allowed to incubate for 30 min. The LOD of the biosensor was found to be 0.01 ng/mL SARS-CoV-2 spike RDB in 30 min.

Liang et al. reported a smartphone-based enzyme-linked immunochromatographic sensor (NLICS) to detect SARS-CoV-2 nucleocapsid protein (NP) in human blood [68]. Au@PtNPs were prepared. For antibody labeling, potassium carbonate (K_2_CO_3_) and detection antibody (mAb1) were added to Au@PtNPs. For N protein detection by NLICS, SARS-CoV-2 N protein was immobilized to Au@PtNPs mAb1 (detection antibody) and mAb2 (capture antibody) sprayed on the membrane. The color formation reaction was catalyzed by a TMB substrate solution. The resulting absorbance was read with a photometer and the results were transferred to a smartphone. Clinical serum samples were collected from COVID-19 patients and an LOD was obtained as 0.026 ng/mL within a linear range of 0.05–1.6 ng/mL in 25 min. The biosensor showed 95.1% accuracy against ELISA kit results.

Liu et al. reported a Raman SERS-LFIA for the detection of anti-SARS-CoV-2 IgM/IgG in clinical samples [69]. To synthesize SiO_2_@Ag core–shell NPs, SiO_2_Au-seed/DTNB NPs were mixed with polyvinylpyrrolidone, AgNO_3_, formaldehyde, and ammonia. The double-layer DTNB-modified SiO_2_@Ag was incubated with S protein via EDC/NHS chemistry, while BSA was used as the blocking agent. For simultaneous detection of anti-SARS-CoV-2 IgM/IgG, the test line was sprayed with anti-human IgM and anti-human IgG. A S protein antibody was added to the control line, whereas the conjugate pad was prepared using SiO_2_@Ag SERS labels. SERS measurements were performed under 785 nm. SARS-CoV-2 S protein antibody was detected within a range of 10–0.001 ng/mL with an LOD of 1 pg/mL. SERS-LFIA has the advantage that it showed rapid result in 1 s with 100% accuracy due to the combined analysis of IgM and IgG. However, the nanoparticle synthesis takes a long time, decreasing the feasibility of the biosensor.

Masterson and Sardar reported epitope-modified plasmic biosensing platforms to detect SARS-CoV 2 IgG antibodies in clinical plasma samples [70]. Glass coverslips modified with gold triangular nanoprisms (Au TNPs) were glued to the wells on the 96-well plate. 96-well nanoplasmic biosensors were incubated with COVID-19-positive patient plasma, and absorbances were collected with a microplate reader at 850 nm. The LOD was found to be 63.6 aM with 90% specificity and 100% sensitivity. In another report, Fu et al. developed Au@Pt nanoparticles for colorimetric detection of SARS-CoV-2 spike protein (Fig. 2B) [71]. After Au@Pt NPs were synthesized and incubated with an S1 detection antibody, it was placed into a 96-well polystyrene plate, followed by blocking with BSA. Afterwards, the wells were incubated with S1 protein. To initiate catalysis, TMB and H_2_O_2_ were introduced to the wells and the reaction was stopped with H_2_SO_4_. Colorimetric detection was based on peroxidase catalysis of Au@PtNPs within 30 min. The absorbance was recorded at 450 nm to determine S1 protein of SARS-CoV-2 and the biosensor exhibited an LOD of 11 ng/mL within the linear range of 10–100 ng/mL.

Song et al. reported a fluorescence biosensor detect for SARS-CoV-2 IgG antibody in serum using optofluidic POC testing [72]. The optofluidic point-of-care testing platform (OPOCT) consists of an optical module, fluidic module, electronic control module, and data processing module. The cell was incubated with S-IgG specifically bound to RBD. The cell was then incubated with fluorescently labeled secondary antibodies. IgG bound to the optical fiber surface by a specific antigen–antibody reaction, followed by the addition of fluorescently labeled secondary antibody. IgG was detected in the range of 12.5–1000 ng/mL with an LOD of 12.5 ng/mL. The recovery rate of the OPOCT biosensor was between 67.78 and 125.59% in serum samples. Sun et al. developed a biosensor capable of both colorimetric and fluorescence detection of SARS-CoV-2 N protein (Fig. 2C) [73]. To produce CoFe/NC@Pt, chloroplatinic acid (H_2_PtCl_6_-6H_2_O) and NaBH_4_ were added to CoFe/NC and dispersed in EDC/NHS to generate CoFe/NC@Pt-Ab_2_ conjugates. 96-well plates were first coated with Ab_1_, and then N protein and CoFe/NC@Pt-Ab_2_ conjugates were added respectively. Co/FeNC@Pt showed peroxidase-like activity and produced color signals with TMB in the presence of H_2_O_2_. The sensor was found to have an LOD of 0.022 ng/mL colorimetrically and 0.018 ng/mL fluorescently in a range of 0.05–100 ng/mL. Artificial saliva and human serum were diluted in PBS and tested. The recovery rates were 94.8–113.5% and 93.5–114.2% for artificial saliva and for human serum, respectively, showing that the dual-mode immunosensor can detect SARS-CoV-2 N protein colorimetrically and fluorescently.

Samavati et al. developed an Au/fiber Bragg grating (FBG) probe decorated with GO (Fig. 2D) [74]. A single-mode glass optical fiber was used for FBG production at 1547 nm, and fluorine-doped silica was used as the coating material. Then, it was coated with Au nanolayer via sputtering technique and decorated with GO via drop-cast. Carboxyl end groups were activated with EDC/NHS to immobilize the glycoprotein S antibody of the COVID-19 virus. The probe was immersed in the patient’s saliva, and LOD was obtained as 1.6 × 10^3^ copies/mL. Kawasaki et al. reported an imprinted photonic crystal film (IPCF)–based optical sensor for label-free detection of SARS-CoV-2 spike protein [75]. IPCF chip made of cycloolefin polymer (COP) was incubated with glutaraldehyde (GA) aqueous solution to immobilize the anti-SARS-CoV-2 spike antibody. Optical measurements were performed using a tungsten-halogen lamp light source with optical fiber and a refractometer equipped with a smartphone and a complementary metal oxide semiconductor (CMOS) sensor. The reflection intensity was proportional to target SARS-CoV-2 spike proteins and the IPCF sensor showed a linear concentration range of 1 pg/mL–100 ng/mL with an LOD of 429 fg/mL for spike proteins in artificial saliva.

Antibody-based biosensors are widely used for the development of optical biosensors due to their high affinity to whole viruses and viral proteins. The working principle of antibodies in biosensors is that they can interact strongly with their specific antigens. While antibodies show high specificity and affinity towards the target analyte, they have some disadvantages such as instability and detection of different epitopes in the same pathogen by polyclonal antibodies [76].

### Antigen-based biosensors

**Fig. 3:**
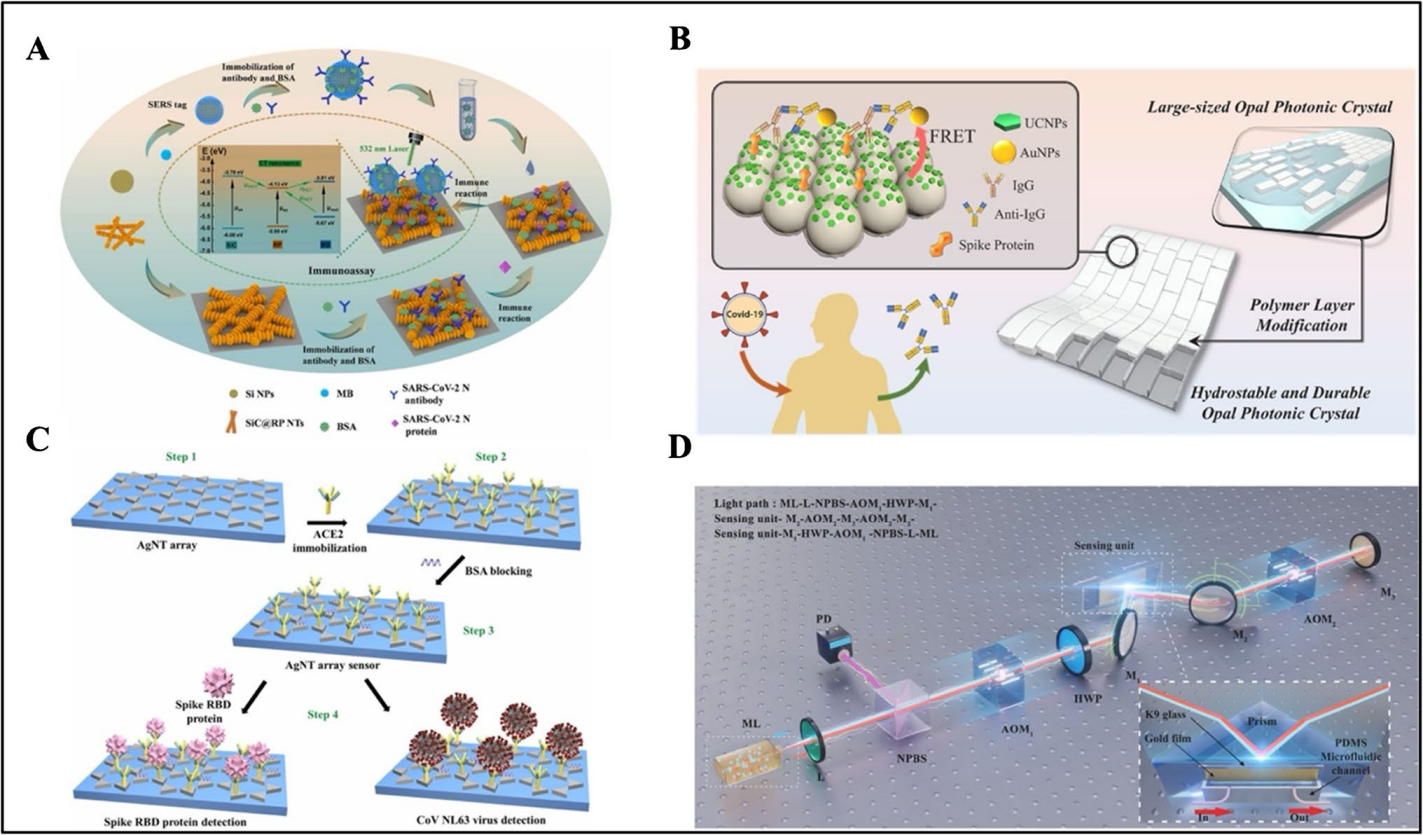
**A** Schematic illustration of the SERS-based immunoassay. Reproduced with permission from Ref. [77]. **B** Schematic illustration of optimized OPC film and subsequent sensing process. Reproduced with permission from Ref. [78]. **C** Proposed strategy for spike RBD protein or virus detection. Step 1: AgNT array fabrication. Step 2: ACE2 immobilization. Step 3: BSA blocking. Step 4: spike RBD protein or CoV detection. Reproduced with permission from Ref. [79]. **D** Schematic diagram of the biosensor. ML, microchip laser; L, lens; NPBS, non-polarization beam splitter; PD, photodetector; AOM_1_, AOM_2_, acousto-optic modulators; HWP, half-wave plate; M_1_, M_2_, M_3_, mirrors. Inset: the schematic diagram of the sensing unit, in which the red arrows represent the in and out of the samples. PDMS, polydimethylsiloxane. Reproduced with permission from Ref. [80]

Liu et al. developed a SERS biosensor modified with silicon nanoparticles and SiC@RP nano-tower (NT) semiconductor substrates to detect SARS-CoV-2 N protein (Fig. 3A) [77]. SiC nanowires (NWs) were prepared by mixing the aerogel structure formed by polycarboxylan and vinyl compound with rice husk carbon and silicon under argon protection. The obtained SiC NWs were separated in hydrofluoric acid and under UV light to obtain SiC NTs. SiC NTs and RP were mixed by heating, and SiC@RP was obtained in the desired ratios. When immunoprobes were produced, methylene blue (MB) was incubated with Si NP solution. Then, target antigen solutions were added to generate a sandwich immunoassay. It was found that bare SiC NTs gave a higher SERS signal than SiC NWs. In addition, SiC@RP, SiC, and RP were compared separately. The peaks of SiC@RP were found to be 2.5 times higher than those of the other two. The sensor was calculated to have a linear range of 1 × 10^−5^ to 1 × 10^−9^ g/mL and a LOD of 7.6 × 10^−11^ g/mL. As a result of the tests with the sandwich immune structure, it was observed that the SERS signals of viruses and proteins other than the SARS-CoV-2 N protein were low, while the SERS signal of the N protein was intense. The biosensor was tested in saliva with a recovery rate of over 80%.

Hu et al. reported an opal photonic crystal (OPC) structure–based coronavirus IgG antibody upconversion FRET biosensor for COVID-19 detection (Fig. 3B) [78]. At first, a stable OPC film was prepared using PMMA microsphere solution in the presence of a mixture of methyl methacrylate (MMA) and K_2_S_2_O_8_. Afterwards, UCNPs were prepared by mixing yttrium triacetate tetrahydrate, ytterbium triacetate tetrahydrate, erbium triacetate tetrahydrate, oleic acid, and octadecene. PEI aqueous solution was then added to the final component to obtain PEI-UCNPs. The OPC film was soaked in dopamine solution for 12 h and PEI-UCNP dispersion solution overnight, respectively, to generate polydopamine (PDA)/OPC film on the UCNPs surface. Therefore, the hybrid fluorescence film was obtained and allowed to incubate with PEI ligands to immobilize coronavirus spike protein in the presence of glutaraldehyde due to the interaction between amino groups of PEI and carboxyl groups of coronavirus spike protein. After the modified film was dipped into IgG samples, Au-conjugated anti-IgG was incubated in the detection area. Photographs of the detection areas were recorded with a smartphone camera under 980-nm laser excitation. The upconversion FRET method showed a linear range between 0.5 and 50 ng/mL concentration with an LOD of 0.092 ng/mL. The biosensor was validated with diluted blood samples and showed a linear range in the concentration range of 0.5–10 ng/mL.

Yang et al. developed a LSPR for the detection of SARS-CoV-2 spike (Fig. 3C) [79]. After titanium and silver NPs were deposited on a glass substrate, polydimethylsiloxane was coated to form silver nanotriangles (AgNT). The LSPR sensor was modified using human angiotensin ACE2, followed by blocking the non-specific regions with the use of BSA. The LOD value of the spike protein was 0.83 pM in 20 min. Furthermore, the biosensor could be implemented using a portable UV–Vis spectrometer. Dai et al. reported a SPR biosensor based on laser heterodyne feedback interferometry for the detection of SARS-CoV-2 spike antigen (Fig. 3D) [80]. PDMS microfluidic channel/gold film/K9 glass sandwich structure was generated on a prism as the sensing unit. At first, gold film (GF) was deposited on K9 glass via the electron beam evaporation (EBE) method, whereas the PDMS microfluidic channel was fabricated by molding and bind to K9 glass. Finally, the prism and sandwich structure were combined. As for biosensor surface modification, the GF on the sensing surface was treated with positively charged poly-L-lysine (PLL) solution and anti-SARS-CoV-2 spike monoclonal antibody (MAbs) was immobilized. Non-specific binding sites were blocked with BSA solution. SARS-CoV-2 spike antigen was detected in a linear detection range from 0.01 to 1000 ng/mL with an LOD of 0.08 pg/mL without sample labeling.

In conclusion, LFIA strips were able to detect the presence of neutralized antibodies in the sample in three modes and this method holds great potential for the future. The studies presented here are summarized in Table 2.

**Table 2:** Antigen-based biosensors

Nanomaterial	Optical method	Linear range	LOD	Real sample	Reference
SiC@RP	SERS	1 × 10^−5^–1 × 10^−9^ g/mL	7.6 × 10^−11^ g/mL	Saliva	[77]
PDA/OPC	Fluorescent	0.5–10 ng/mL	0.092 ng/mL	Blood sample	[78]
Ag NPs	LSPR	-	0.83 pM	-	[79]
PDMS	SPR	0.92–1000 ng/mL	0.08 pg/mL	SARS-CoV-2 spike antigen samples	[80]

Antigen-based tests are more often used in saliva than blood. Antigens are less likely to be degraded during their transportation compared to other biomarkers, ensuring good results in the long term [81]. Negative aspects of the method are that there are limited studies on developing antigen tests and there is low availability of test kits on the market [82].

### Nucleic acid–based biosensors

SARS-CoV-2 possesses a single-stranded RNA genome of approximately 30 kilobases in length [9]. As mentioned previously, the genome is coated with the viral nucleocapsid protein, which protects and condenses the viral genome. Like any RNA virus, the SARS-CoV-2 genome is comprised of a specific sequence of nucleobases (adenine, cytosine, thymidine, and uracil), and nucleic acid probes can be quickly and inexpensively developed against the specific viral RNA sequences that can be used on biosensors to detect viral genomes. SARS-CoV-2 is commonly detected using reverse-transcription polymerase chain reaction (PCR) assays (real-time PCR or digital droplet PCR) to detect viral genomes in patient samples, which provides highly specific and sensitive detection of viral genomes in a short time period [83].

While the detection of viral nucleic acids is a powerful method for diagnosis, there are a number of challenges that need to be addressed for effective detection with biosensors. Before viral RNA can be detected, it must first be extracted from either viral particles or infected cells present in patient samples. Extraction of viral RNA from patient samples for standard PCR testing is usually accomplished using chemical extraction to dissolve lipid membranes and denaturate viral proteins coating the RNA genome, selective purification of viral RNAs, followed by reverse transcription to generate stable DNA versions of the unstable viral RNA. Without an effective sample preparation, viral genomes cannot be detected as the genome will be unavailable to bind to the nucleic acid probe in the diagnostic assay. Another issue with nucleic acid detection is the presence of secondary structures within single-stranded RNA virus genomes that may block nucleic acid probe binding sites [84, 85]. Naturally occurring secondary structures can form rapidly due to intramolecular folding, and if probes are not designed carefully towards regions with limited or no secondary structure, heat-mediated denaturation and annealing of the viral genome in the presence of probes is generally required for probe:genome hybridization and viral genome detection. While heat-mediated hybridization can be quite effective (as is seen with PCR denaturating/annealing steps), it requires the sample to either be annealed to the probe before being added to the biosensor or the biosensor to be robust enough to tolerate temperature cycling approaching 100 °C. Another key challenge for nucleic acid biosensors is viral genome mutation. The viral genome sequence can mutate in response to selective pressures (replication changes, immunological pressures, etc.), and synonymous mutations that change the nucleotide sequence of the genome while maintaining the amino acid sequence emerge rapidly across viral quasispecies, altering the ability of viral genomes to be detected by nucleic acid–focused diagnostic assays. The evolution of SARS-CoV-2 throughout the current pandemic clearly demonstrates the challenges with the detection of viral RNA genomes [83]. The rapid evolution of variants of concern (VOC) from the initial outbreak through the delta and now omicron lineages [86] has made the detection of SARS-CoV-2 a moving target over the last several years, requiring diagnostic assays to change in response to circulating lineages.

Aptamers are short, single-stranded DNA or RNA (ssDNA or ssRNA) molecules that can selectively bind to a specific target, which have been selected via a process called SELEX for their ability to bind to specific molecular features of antigens. A primary factor in the appeal of aptamers is that they can be generated in quantity via well-developed chemical synthesis procedures at low cost and do not require animals to be generated. Aptamers can be derived from single-stranded DNA molecules, RNA molecules, or modified nucleotides incorporated into oligonucleotide sequences. Randomized library oligonucleotides are incubated with purified target antigen, and aptamer sequences that are able to bind to the antigen are isolated, amplified, and used in subsequent rounds of selection to identify aptamers that bind specific antigen sequences with high specificity and sensitivity. A number of aptamers have been identified that bind to SARS-CoV-2 antigens [87–90]. Sensors developed using aptamers have many advantages over antibody-based sensors, including the ability to be engineered, high affinity, and high selectivity although affinity and selectivity are generally not as high as found with antibodies.

**Fig. 4:**
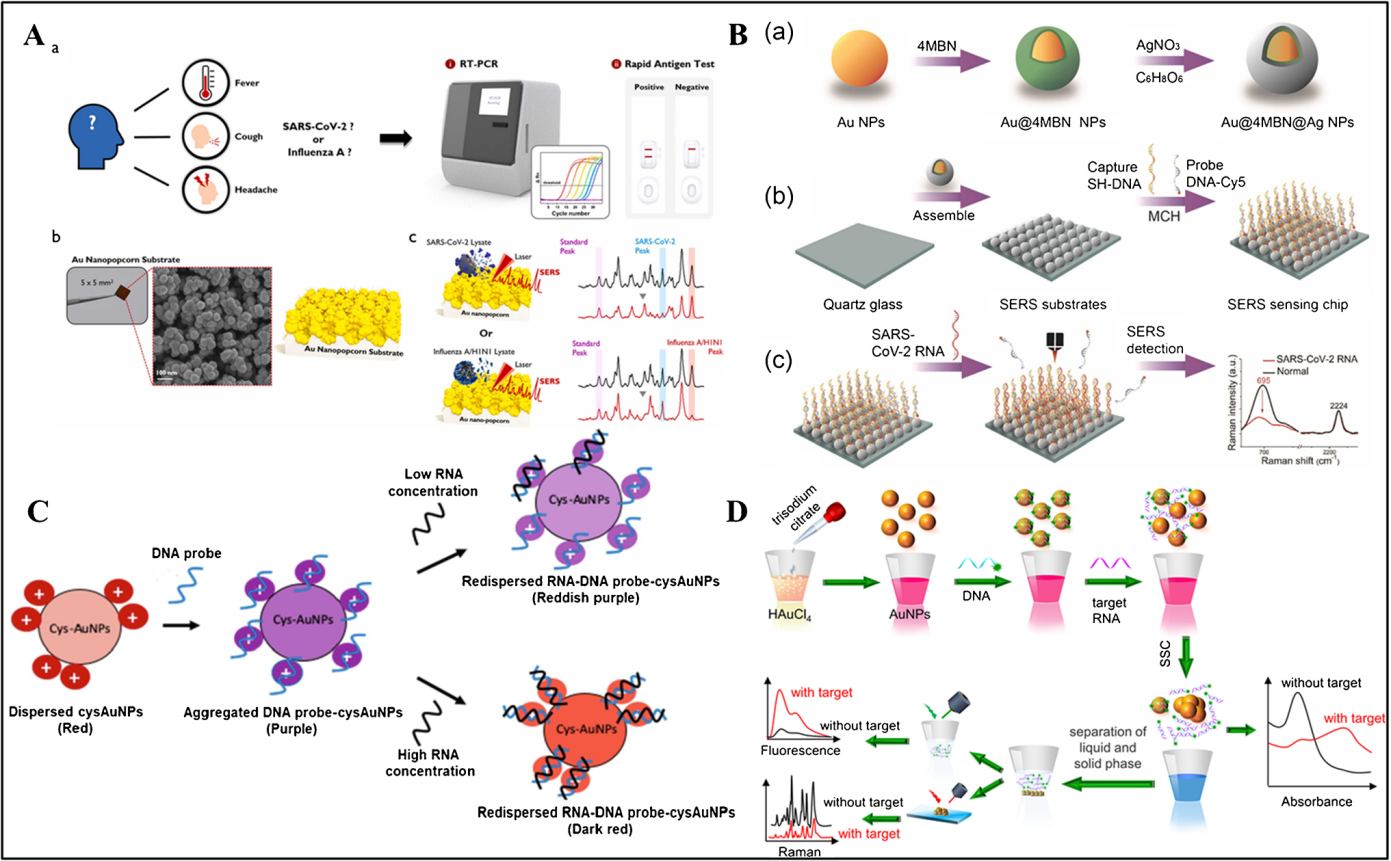
**A** (a) Symptoms that appear when infected with SARS-CoV-2 or influenza A and RT-PCR and rapid antigen kit for their diagnosis. (b) Photograph and SEM image of Au nanopopcorn substrate. (c) Working principle of dual aptamer-immobilized Au nanopopcorn substrate for virus assays. Reproduced with permission from Ref. [91]. **B** Schematic illustration of (a) the fabrication of Au@ 4MBN@Ag NPs, (b) the preparation of a two-dimensional SERS sensing chip, and (c) the SERS sensing chip for detection of SARS-CoV-2 RNA. Reproduced with permission from Ref. [92]. **C** Schematic representation of the working principle of the proposed reflectometric-based RNA biosensor based on cysAuNP colorimetric agent for optical detection of target RNA. Reproduced with permission from Ref. [93]. **D** Schematic of the triple-mode biosensors for COVID-19 virus RNA detection. Reproduced with permission from Ref. [96]

The nucleic acid–based nanomaterial-modified biosensors demonstrated here are presented in Table 3. Chen et al. developed a dual-mode SERS sensor that can simultaneously detect SARS-CoV-2 and influenza A/H1N1 viruses (Fig. 4A) [91]. Au popcorns were formed on a PET suing conventional sputtering and thermal evaporation methods. For functionalization, capture DNA and aptamer probes for each corresponding virus were incubated separately. The DNAs were interacted with tris(2-carboxyethyl) phosphine hydrochloride (TCEP) to activate the thiol groups. To enhance SERS signals, cyanine (Cy3) reporter was added to the spike protein DNA aptamer, whereas Rhodamine Red™-X (RRX) reporter was added to the H1N1 capture DNA aptamer. The amount of virus was determined due to the decrease in peak intensity of Raman reporters. The LOD value was determined as 0.78 PFU/mL for SARS-CoV-2, whereas it was determined as 0.62 HAU/mL for H1N1 samples. The SERS biosensor has the advantage that it can be operated in dual-mode for the detection of SARS-Cov-2 and influenza A. However, it should be confirmed by testing in the real samples. In another report, Lin et al. developed a two-dimensional ratiometric SERS detection chip with a core-molecule-shell-molecule nanostructure to detect SARS-CoV-2 RNA (Fig. 4B) [92]. The developed SERS chip consists of an interference-free internal standard (IS) molecule and a probe molecule (Cy5). Au NPs were first synthesized and mixed with 4-mercaptobenzonitrile (4MBN) to obtain Au@4MBN NP colloid. Then, ascorbic acid and silver nitrate solutions were added to the mixture to form Au@4MBN@Ag NPs. The final product was added to a quartz glass surface. Then, SH-DNA and probe DNA-Cy5 were added to the SERS chip to detect SARS-CoV-2 RNA on the SERS substrate in 20 min. The working principle of the sensor was based on the interaction between SARS-CoV-2 RNA and the probe DNA, resulting in a decrease of Raman signal. The LOD was obtained as 7.61 × 10^−14^ M in the linear detection range of 10^−6^–10^−12^ M. However, the stability of the biosensor was 7 days, which should be improved further in the future.

**Table 3:** Nucleic acid–based biosensors

Nanomaterial	Optical method	Linear range	LOD	Real sample	Reference
Au nanopopcorn	SERS	-	0.78 PFU/mL	-	[91]
Au@4MBN@Ag NPs	SERS	10^−6^–10^−12^ M	7.61 × 10^−14^ M	Saliva	[92]
cysAuNPs	Reflectometric	25–200 nM	0.12 nM	RNA samples of the oropharyngeal swab from COVID-19 patients	[93]
AuTNPs	LSPR	1.56–312 ng/mL	0.035 nm/(ng/mL)	-	[94]
AgNCs	Fluorescence	0.30–10.0 nM	0.30 nM	Clinical samples	[95]
Au NPs	Colorimetric–fluorescence–SERS	-	Colorimetric: 160 fM Fluorescence: 259 fM SERS: 395 fM	Simulated samples	[96]
IMSN	Colorimetric	-	240 copies/mL	Oropharyngeal swab	[97]
AuNPs/COFs	SERS	1 × 10^−6^–1 ng/mL	2.7 × 10^−7^ ng/mL	Serum–saliva	[98]
Au/PET	SERS	0–1000 PFU/mL	3.67 PFU/mL	Nasopharyngal swab	[99]
Au NPs	SERS	250–10000 TU/μL	124 TU/μL	Oropharyngeal swab	[100]
RCA-Au NPs	LSPR	-	148 vp/mL	Pseudovirus	[101]
Nb_2_C-SH QD	SPR	0.05–100 ng/mL	4.9 pg/mL	Human serum	[102]
CTs	Fluorescence	100 fg/mL–1 μL/mL	89.7 fg/mL	-	[103]
Au PIAF	Fluorescence	0.1–200 pg/mL	65 fg/mL	Saliva	[105]

Jamaluddin et al. developed an optical reflectometric–based cationic cysteamine-capped gold nanoparticles (cysAuNPs) for the detection of COVID-19 RNA (Fig. 4C) [93]. CysAuNPs were generated using HAuCl_4_ and cysteamine. NaBH_4_ was then added to the solution, and the single-stranded DNA probe was incubated for 5 min. The target RNA was then incubated on the DNA probe modified with cysAuNPs for 30 min, resulting in visual color change due to the competitive binding of the DNA probe to the cysAuNPs and target RNA. This biosensor showed a linear range of 25–200 nM with an LOD of 0.12 nM in 30 min. The visualized color change could be performed with portable devices, such as smartphone camera or scanner devices to obtain measurement results. In addition, it is possible to quantify COVID-19 RNA with reflectance spectra intensities via a spectrometer coupled to a bifurcated optical fiber with a UV–Vis-near-infrared (NIR) light source in the wavelength range of 200–1099 nm. This biosensor has the advantage that it is capable of diagnosing COVID-19 disease including asymptomatic carriers with low viral load even in the presence of co-infection with other viruses without complex instrument. However, DNA hybridization reaction takes 30 min leading to longer analysis time.

Xu et al. developed an optical fiber LSPR sensor based on the detection of Au TNPs for SARS-CoV-2 [94]. To biofunctionalize the optical fiber surface, the fiber probe was treated with hydrochloric acid and methanol solution to activate the hydroxyl group. The dried fiber was treated with MPTMS-ethanol solution to convert the hydroxyl group of the probe into the sulfhydryl group. Afterwards, chloro(triethyl phosphine) was mixed with gold (I) and acetonitrile for AuTNP synthesis. The optical fiber surface was coated with AuTNP and then activated with MUA and EDC/NHS, respectively. The light entered through the Y-type optical fiber, generating localized surface plasmon resonance phenomenon with the AuTNP material on the probe surface, whereas the LSPR spectrum was shifted to blue for quantification of SARS-CoV-2 spike antigen in 10 min within a linear range from 1.56 to 312 ng/mL.

Molaabasi et al. reported an amplification-free ratiometric fluorescent biosensor for COVID-19 from isolated RNA samples [95]. After the DNA template was incubated with AgNO_3_ solution, NaBH_4_ solution was added to the resulting DNA/Ag^+^ mixture. Graphite powder, KMnO_4_, and H_2_O_2_ were mixed to obtain GO. Then, DNA-silver nanoclusters (AgNCs) were diluted and mixed with GO suspension. COVID-19-positive and negative throat swab samples were collected and processed with a commercial kit (KBC) for total viral RNA extraction and purification. Fluorescence spectra of DNA-AgNC/GO nanohybrids under blue and green excitation wavelengths were recorded. By adding viral RNA to nanohybrid fluorescent probes, the difference in response between negative and positive samples was detected and LOD was obtained as 0.30 nM, linear range was obtained as 0.30 to 10.0 nM in 12 min. The specificity and sensitivity of the probe is > 90%. The biosensor offers the use of a small sample volume of 5 μL.

Gao et al. developed a DNA-based biosensor that can simultaneously detect SARS-CoV-2 RNAs in a colorimetric/fluorescence/SERS triplet (Fig. 4D) [96]. At first, AuNPs were obtained by mixing chloroauric acid with a trisodium citrate solution. Then, DNA probes were then incubated with AuNP solutions. The target RNAs were added to this mixture, followed by the addition of saline sodium citrate (SSC) for aggregation induction. When DNA probes were mixed with AuNPs and interacted with the target RNA, changes in the concentration of RNA and absorbance peak occurred. The biosensor had an LOD of 0.58 pM in absorbance, 1.11 pM fluorescence, and 2.17 pM SERS signal. The sensor had the advantage that it showed long stability for 18 months. The sensor was applied to simulated samples of target RNA added to TE buffer (10 mM Tris–HCl and 1 mM EDTA) with recoveries of 97–126%. Accordingly, it was determined to have an LOD value of 160 fM, 259 fM in fluorescence and 395 fM in SERS for real samples.

He et al. developed colorimetric detection of SARS-CoV-2 nucleic acid using iron manganese silicate nanozyme (IMSN) [97]. Due to the peroxidase (POD)–like action of IMSN, they used a detection technique based on the suppression of pyrophosphate ions (PPi) created by amplification in the sensor. To prepare IMSN, dendritic mesoporous silica nanoparticles (DMSN) were first prepared by the two-phase stratification method. Then, it was mixed with manganese chloride, ferrous sulfate heptahydrate, and ammonium chloride solution for 12 h and then dried at 60 °C overnight to complete the production step. IMSN and TMB as substrates were added to the H_2_O_2_ buffer system at varying concentrations and absorbance changes were evaluated. According to the results, IMSN showed POD-like behavior by catalyzing the color change in TMB in a good way. A trap probe was used in an electron spin resonance assay to find the presence of ^−^OH, which is produced when IMSN catalysis decomposes H_2_O_2_. With the addition of PPi to the medium, ^−^OH could not be detected. This result suggests that PPi may inhibit POD-like activity in cooperation with iron and manganese ions. It was found that IMSN gave good results in the range of pH 3–5 and temperatures 20–60 °C, which is suitable for routine testing. For SARS-CoV-2 detection, the DNA of the ORF1ab gene was amplified and tested. IMSN was able to detect the target up to 240 copies/mL. In positive samples, all genes formed excess amounts of PPi. On the contrary, in negative samples, inhibition of IMSN was observed only in the RNase P gene. At the end of these treatments, all samples showed color changes visible to the naked eye. This method has potential not only for the SARS-CoV-2 virus but also for the detection of other viruses.

**Fig. 5:**
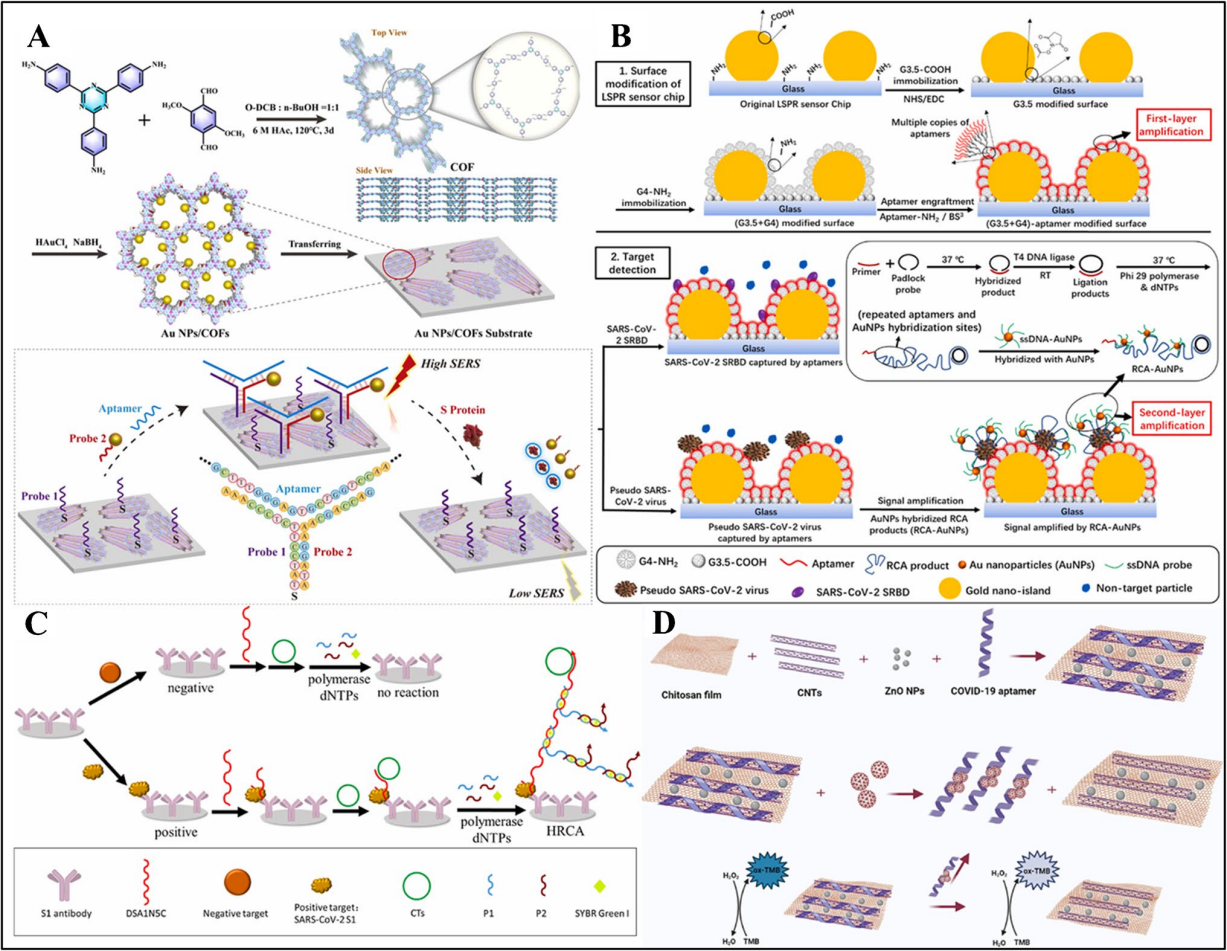
**A** Schematic illustration of the synthesis of AuNPs/COF substrate, and the SERS detection of S protein based on the Y-shaped aptasensor. Reproduced with permission from Ref. [98]. **B** Schematic illustration showing the general approach to prepare (G3.5 + G4) aptamer-modified LSPR sensor chips for sensitive detection of the SARS-CoV-2 SRBD and SARS-CoV-2 pseudo viral particles. Note that the second-layer amplification is only possible with SARS-CoV-2 pseudo viral particles as the detection targets upon which the detection sandwich format can be built. Reproduced with permission from Ref. [101]. **C** Schematic illustration of the HRCA-based aptasensor for SARS-CoV-2 S1 protein. Reproduced with permission from Ref. [103]. **D** Schematic representation of proposed platform for detection of COVID-19. Reproduced with permission from Ref. [106]

Xie et al. developed a Y-shaped aptasensor for SARS-CoV-2 S protein detection based on composites of covalent organic frameworks doped with gold nanoparticles (AuNPs/COFs) (Fig. 5A) [98]. COFs were first synthesized using 2,4,6-tris-(4-aminophenyl)triazine (TAT) and 2,5-dimethoxy-terephthalaldehyde (DMTA) in o-dichlorobenzene, n-butanol, and acetic acid by heating for 3 days. Then, chloroauric acid was added to COFs to obtain AuNP/COFsNPs. The Y-shaped structure consists of three parts including thiolated probe 1, conjugated Au-4-aminothiophenol-silver-gold nanoparticles (Au@4-ATP@Ag@AuNPs), and aptamer probe 3 against the S protein. AuNP/COF composites on probe 1 and Au@4-ATP@Ag@Au NPs on probe 2 were prepared by deposition. This structure was used as a SERS substrate, and S protein detection was performed according to the principle of decreasing the SERS signal in the presence of S protein. The analysis showed that the addition of probe 2 and an aptamer on probe 1 substrate generated strong SERS signals, indicating that the Y-shaped aptasensor was successfully coupled. The LOD of the sensor was 2.7 × 10^−7^ ng/mL in the linear range of 1 × 10^−6^ to 1 ng/mL. Measurements with S protein added to serum and saliva collected from the healthy volunteers showed a decrease in SERS intensity and accurate detection with recoveries between 96 and 105%.

Chen et al. developed an aptamer-based SERS biosensor for detecting SARS-CoV-2 lysates [99]. A protective polyethylene PET polymer substrate was used as the substrate and coated with Au layer. Then, 1H,1H,2H,2H,2H-perfluorodecanethiol (PFDT) solution was poured into a petri dish and attached to the Au/PET substrate. Au was deposited on the substrate by thermal evaporation to obtain the Au popcorn surface. The substrate was then incubated with a DNA aptamer capture solution, followed by immersing it in a 6-mercapto-1-hexanol (MCH) solution containing 4MBA to increase the specificity of the biosensor. The thiol groups of DNAs were activated with TCEP, and Raman reporter Cy3 was added to the capture DNA. Virus lysates were drop-cast on the Au nanopopcorn substrate for analysis. Tests were performed with SARS-CoV-2 lysates at concentrations ranging from 0 to 1000 PFU/mL with an LOD of 0.53 PFU/mL. Clinical tests were performed with SARS-CoV-2 lysates added to negative nasopharyngeal swabs and the LOD value was found to be 3.67 PFU/mL. SERS detection of SARS-CoV-2 using the spike protein aptamer showed twofold better sensitivity than commercialized SARS-CoV-2 kits, demonstrating a potential for sensitive detection of SARS-CoV-2. Guan et al. developed a one-step SERS biosensor based on aptamer recognition to detect the SARS-CoV-2 spike protein [100]. At first, streptavidin-modified magnetic beads were mixed with biotin and conjugated with S protein to obtain the spike protein aptamer-conjugated magnetic beads. To prepare SERS nanoprobes, AuNPs and Nile blue A (NBA) Raman reporter were mixed, followed by conjugation of the thiolated spike protein aptamer mixture to the AuNPs surface. The remaining regions on the surface were blocked with SuperBlock Blocking Buffer (TBS). After aptamer-conjugated magnetic beads and aptamer-conjugated SERS nanoprobes were resuspended, the sandwich mixtures were separated with a magnetic rod. LOD of the biosensor in the linear range of 250–10000 TU/μL was determined as 124 TU/μL with a sensitivity of 95% in 40 samples from both negative and positive samples.

Hao et al. developed LSPR sensor chips based on SARS-CoV-2 aptamer poly(amidoamine) (PAMAM) dendrimers (Fig. 5B) [101]. To fabricate LSPR sensor chips, poly(allylamine hydrochloride) (PAH) and poly(sodium 4-styrene sulfonate) (PSS) were used as substrates and modified with AuNPs. MUA was used to generate carboxyl functional groups on the electrode surface. The fabricated LSPR sensors were modified with generation 3.5 (G3.5) carboxylated PAMAM dendrimers to avoid surface contamination and generation 4 (G4) aminated PAMAM dendrimers, which serve as multi-arm tags for subsequent conjugation of aptamers sensitive to bind spike protein receptor of SARS-CoV-2. Then, BSA was used to prevent non-specific adsorption. Signal detection was enhanced by using rolling circle amplification (RCA)-AuNP as a second-layer amplification. ssDNA-AuNP and RCA conjugates were incubated at 37 °C for 30 min and RCA-AuNP complex was obtained. The spike RBD was used to evaluate the aptamer biosensor, and the LOD value was found to be 21.9 pM. As a secondary target, SARS-CoV-2 pseudoviral particles were utilized. When this target was tested, it was observed that the RCA-AuNP combination produced weak signals before application but substantially stronger signals after application, showing an LOD of 148 viral particles (vp)/mL. The signals in the SARS-CoV-2 SRBD were found to be significantly stronger than the others, demonstrating that the biosensor is highly specific to SARS-CoV-2. SARS-CoV-2 SRBD and SARS-CoV-2 pseudoviral particles showed similar detection characteristics in synthetic saliva, which shows its capability in accurate detection in complex biomatrixes.

Chen et al. developed a SPR aptasensor based on niobium carbide MXene quantum dots (Nb_2_C-SH QD) for the detection of SARS-CoV-2 N protein [102]. At first, Nb_2_C MXene was obtained by drying Nb_2_AlC powder to prepare Nb_2_C QDs. Nb_2_C MXene in water was heated in an autoclave at 100 °C and filtered through a 220-nm membrane to prepare Nb_2_C QDs. Nb_2_C-SH QDs were then obtained by adding n-octadecyl mercaptan. A commercial Biacore X100 device was used as an SPR working chip. The synthesized Nb_2_C-SH QDs were used to modify gold chip surface, followed by immobilization of commercial N58 aptamer via π-π* stacking, electrostatic adsorption, and hydrogen bond. Within 1000 s, a concentration-dependent increase in the range of 0.05 to 100 ng/mL with an LOD of 4.9 pg/mL was observed. Human serum, seawater, and seafood were used to test the biosensor and the recovery rates were 98.67–104.86%, 97.70–111.30%, and 91.80–95.86%, respectively.

Wang et al. developed a fluorescent aptasensor based on hyperbranched rolling cycle amplification (HRCA) for the detection of SARS-CoV-2 S1 protein and pseudovirus (Fig. 5C) [103]. When preparing circular templates (CT), padlock and complementary DNA (CDNA) were incubated in T4 DNA ligase reaction buffer. CT was prepared by adding exonuclease I and III to the solution for 4 h. 96-well plates were coated with antibodies in coating buffer and blocked with BSA for 2 h. Due to the formation of antibody-aptamer sandwich complexes, aptamers were synthesized by systematic evolution of ligands by exponential enrichment (SELEX) method. SYBR Green 1 was added as a double-stranded DNA fluorescence indicator to generate a fluorescent signal [104]. Then, fluorescence intensity was found to be at 525 nm due to the formation of S1-aptamer-CT sandwich complex by adding SARS-CoV-2 spike antibody, DSA1N5C aptamer, and CTs to 96-well plates via physical adsorption method. The aptasensor had an LOD of 89.7 fg/mL within a linear range of 100 fg/mL–1 μL/mL. The aptasensor was tested in influenza haemagglutinin peptide fragment (HA1), His (a tag on the S1 protein), human serum albumin (HSA), BSA, and artificial saliva. It was found that HA1, His, and artificial saliva did not give a significant fluorescence signal, whereas BSA gave a low signal and HSA gave half the signal seen in the S1 protein. This indicates that the aptasensor has a good selectivity for S1 protein. Although the aptasensor has the potential for early detection of SARS-CoV-2 and pseudovirus, the sensitivity and selectivity of the sensor should be investigated in real samples.

Zhu et al. developed an aptamer-based biosensor using in-frame gold particle nanostructures (Au PIAFs) as metal-enhanced fluorescent material to detect SARS-CoV-2 N protein [105]. Au PIAFs were synthesized with the use of Ag prism seeds in the presence of a mixture consisting of AgNO_3_, sodium citrate, PVP, H_2_O_2_ and NaBH_4_, potassium iodide (KI), and hydrogen tetrachloroaurate(III) trihydrate (HAuCl_4_-3H_2_O). Then, Apt 1 and Apt 2 aptamer solutions were added to the synthesized Au PIAF. The carboxyfluorescein (FAM) fluorophores in Apt 1 provide an important amplification of the fluorescence signal. For N protein detection, the N protein sample was added to the Au PIAF@Apt (APA) biosensor, and then fluorescence intensity was analyzed. The study was performed with an internal reflection fluorescence (TIRF) microscope under 488-nm laser beam excitation, and the samples were recorded with a camera and analyzed with software in 3 min. The LOD value was 44 fg/mL in the linear range of 0.1–200 pg/mL, whereas it was 65 fg/mL with a recovery rate of 97.10–103.3% in human saliva. Environmental swabs such as door handles and faucets were also collected and analyzed against commercial antigen kits with good agreement. It was observed that the sensor produced stable signals even 7 days after annealing with aptamers.

Vafabakhsh et al. developed a paper-based aptasensor using a chitosan film (ChF)/ZnO/carbon nanotube (CNT) nanohybrid (Fig. 5D) [106]. At first, ZnO NPs were synthesized using PEG and zinc sulfate heptahydrate (ZnSO_4_ 7H_2_O), followed by titration with NH_4_OH. Then, ZnO NPs and CNT were dispersed in a chitosan solution for 6 h. The paper-based platform was coated with nanohybrids, and a TMB substrate was added to the paper surface. COVID-19 aptamer was then drop-cast on the spot region of the paper device. After H_2_O_2_ was introduced to the detection spot, blue color was observed due to the enzymatic activity. The resulting color was analyzed with Image J software and the paper-based biosensor showed a linear range from 1 to 500 pg/mL with an LOD of 0.05 pg/mL.

Nucleic acid–based biosensors are advantageous due to their fast analysis times, low sample requirements, and the specific nucleotide sequence of DNA or RNA. In addition, protein binding can be controlled and biosensors that can recognize the target protein can be obtained [107]. One of the disadvantages of this method is the easy degradation of the samples during transportation. In addition, viruses are constantly mutating, making this method less effective for virus detection compared to other biomarkers [81]. Owing to the self-amplification of aptamers, the sensitivity of biosensors increases. Aptamers are able to rapidly and reversibly change their conformation, which can be used to achieve the sensitivity and selectivity needed for real-time detection [108]. Despite these advantages, aptamer-based biosensors could have difficulty in multi-target analysis and relatively high cost and require large sample volumes [109].

### Other sensors

**Fig. 6:**
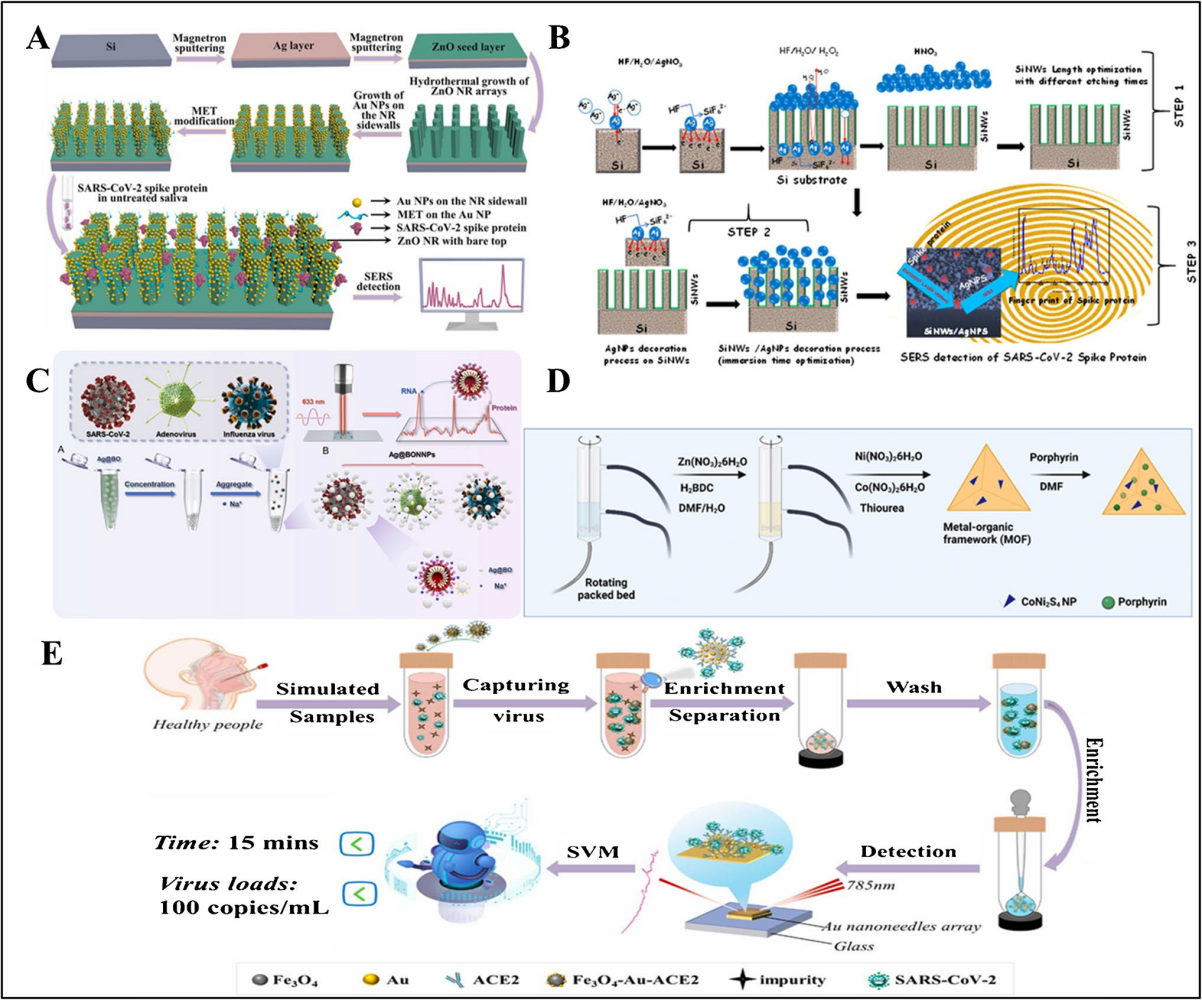
**A** Illustration for the SERS substrate fabrication. The fabrication process involves (i) deposition of an Ag film and a ZnO seed layer on a silicon (Si) wafer, (ii) growth of the vertically aligned ZnO NR arrays on the seed layer, (iii) selective decoration of the Au NPs on the sidewalls of the ZnO NRs, and (iv) MET-modification of the Au NPs’ surface. Reproduced with permission from Ref. [110]. **B** Graphical illustration of the fabrication and fast optical detection of the spike protein of the SARS-COV-2 using SiNWs/AgNPs nanohybrid–based sensors. Reproduced with permission from Ref. [111]. **C** (A) Schematic presentation of the hot spots generated by aggregating sodium borohydride-induced silver nanoparticles. Ag@BO: Silver nanoparticles were obtained by reducing sodium borohydride. (B) Schematic diagram of the virus in the hot spots. Ag@BONNPs: The enhanced substrate formed by adding Na ^+^ to the silver nanoparticles produced by sodium borohydride reduction. Reproduced with permission from Ref. [112]. **D** Schematic illustration of the one-pot synthesis of MOF-5/CoNi_2_S_4_ nanocomposites and nanomaterial fabrication for recombinant SARS-CoV-2 spike antigen assay. Reproduced with permission from Ref. [113]. **E** The schematic diagram of SARS-CoV-2 virus detection process. Reproduced with permission from Ref. [114]

There are few studies on developing sensors for the detection of SARS-CoV-2, in which there is no biorecognition elements used. These works are presented in Table 4. For example, Zhang et al. reported an aptamer and antibody-free SERS biosensor for SARS-CoV-2 spike protein detection in saliva (Fig. 6A) [110]. Ag film and ZnO layer were deposited on the silicon substrate, respectively. The obtained substrate layer was immersed in the solution obtained by mixing zinc nitrate hexahydrate (ZNH) and hexamethylenetetramine (HMT) solutions to grow ZnO NRs. Then, ZnO NRs were dipped into gold (III) chloride and sodium borohydride, respectively to generate AuNPs. The principle of the biosensor was based on a system in which virus detection is performed by adding 2-mercaptoethanol (MET)–modified AuNPs to the sides of zinc oxide nanorods (ZnO NR) grown vertically on a silver (Ag) surface. The LOD of the sensor was 3.6 × 10^−17^ M and 1.6 × 10^−16^ M in PBS and saliva, respectively. The sensor has the advantage that it does not require antibodies and aptamers as biorecognition elements, reducing the cost of analysis. Daoudi et al. developed a silicon nanowire (SiNW) and Ag NP–based SERS biosensor for SARS-CoV-2 S protein detection (Fig. 6B) [111]. At first, SiNWs were generated by immersing the Si layer in a Teflon container containing nitric acid, acetic acid, and hydrogen fluoride. Then, it was immersed in a mixture of silver nitrate and hydrogen fluoride to form SiNW/AgNPs. The interaction between the Ag NPs on the sensor surface and the nitrogen atoms of the spike protein resulted in SERS peaks. The sensor was tested for SARS-CoV-2 S protein with the SERS peak at 1609 cm^−1^ at different concentrations ranging from 9.3 × 10^−6^ to 9.3 × 10^−12^ M with an LOD of 9.3 × 10^−12^ M. However, the biosensor should be tested with clinical samples.

**Table 4:** Other sensors

Nanomaterial	Optical method	Linear range	LOD	real sample	Reference
Au NP	SERS	-	1.6 × 10^−16^ M	Saliva	[110]
SiNW/AgNPs	SERS	9.3 × 10^−6–^9.3 × 10^−12^ M	9.3 × 10^−12^ M	-	[111]
Ag@BON NP	SERS	-	10 PFU/mL	Saliva–serum	[112]
MOF-5/CoNi_2_S_4_	Fluorescence	-	5 nM	-	[113]
Fe_3_O_4_-Au nanocomposite	SERS	-	276 copies/mL	-	[114]
COVID 19-PEB	Fluorescence	-	52.5 pmol	Nasopharyngal swab	[115]
tris-NTA	SPR	-	0.058 µg/mL	Blood samples	[116]
Au–Ag HNSs	Colorimetric SERS	20–1500 ng/mL	20 ng/mL	-	[117]

Zhang et al. developed a SERS biosensor for label-free detection of SARS-CoV-2 (Fig. 6C) [112]. The silver nitrate solution was added to the sodium borohydride solution and mixed. Then, silver sol, virus sample, and sodium borohydride were added to acetonitrile to obtain Ag@BON NP. The SERS substrate was prepared by mixing silver nitrate and sodium borohydride solution. The sodium borohydride replaced the citrate root and reduced silver nanoparticles, resulting in increased SERS signals. In addition, sodium borohydride reduced the silver nanoparticles and the amino group of the spike protein interacted with the metal cation nanoparticles at the hot spot, resulting in a SERS peak. LOD of the sensor was found to be 10 PFU/test. It was observed that SERS signals increased with increasing concentrations of SARS-CoV-2. It was observed that the saliva and serum samples containing the virus showed no deterioration in their SERS signals when they were heated up to 56 °C, demonstrating that the virus remains stable against temperature. In addition, the SERS signals exhibited the range of 826 and 1168 cm^−1^, 741–871 cm^−1^, and 1049–1100 cm^−1^, and 960–1221 cm^−1^ and 1103–1167 cm^−1^ for human adenovirus type 7 (HAdV7), SARS-CoV-2, and H1N1 viruses, respectively. This SERS biosensor allows for multiplexed detection of different viruses due to its characteristic peaks. Thus, the biosensor has an important potential for the diagnosis of viral infections in real patients.

Rabiee et al. reported a metal–organic framework (MOF)–based sensors for the detection of recombinant SARS-CoV-2 spike antigen (Fig. 6D) [113]. For the one-pot synthesis of MOF-5/CoNi_2_S_4_, Zn(NO_3_)_2_6H_2_O, and terephthalic acid (H_2_BDC) were mixed in dimethylformamide (DMF). After the resulting mixture was transferred to the rotating packed bed (RPB) system at a high gravity factor of 182, Ni(NO_3_)_2_.6H_2_O, Co(NO_3_)_2_.6H_2_O, and thiourea were added. Porphyrin (H_2_TMP) was added to the surface of the synthesized MOF. Detection was achieved by exposure to different weights of H_2_TMP and recombinant SARS-CoV-2 antigen incorporated into the sensor surface. Then, all nanomaterials were sterilized with ultraviolet radiation and then incubated with MTT (3-[4,5-dimethylthiazol-2-yl]-2,5-diphenyltetrazolium bromide). Finally, optical absorbance was measured with a microplate reader at 570 nm. The biosensor showed an LOD of 5 nM recombinant SARS-CoV-2 spike antigen.

Li et al. developed a SERS biosensor composed of iron (III) oxide (Fe_3_O_4_)-Au nanocomposites to detect SARS-CoV-2 (Fig. 6E) [114]. After Fe_3_O_4_ was synthesized, Au NPs and APTMS were added to the mixture, followed by collecting the nanocomposite with a magnet. Then, the substrate was incubated with ACE2 labeled Histidine (His-ACE2). For SARS-CoV-2 detection, nose and throat swab samples were incubated with a Fe_3_O_4_-Au-ACE2 modified sensor. The sensor was first tested on SARS-CoV-2 pseudovirus and was found to have a sensitivity of 276 copies/mL in 15 min. However, it was observed that the difference in Raman intensity was due to the ACE2 deformation. The biosensor was found to classify positive samples with full accuracy, showing great potential to detect SARS-CoV-2 in real samples. Kang et al. developed a human angiotensin-converting enzyme 2 (hACE2) mimetic peptide marker (COVID19-PEB) using the FRET method for the detection of SARS-CoV-2 [115]. COVID19-PEB was synthesized from hACE2 mimetic peptide and two oligomers, acceptor and transmitter. It is observed that fluorescence quenching occurs as a result of the FRET effect. The LOD value of COVID19-PEB was determined as 52.5 pmol.

Dong et al. developed a four-channel SPR biosensor capable of simultaneous detection of anti-SARS-CoV-2 S1 antibody [116]. For the multi-channel SPR device, capture molecules were immobilized into different channels covering the surface of nitrilotriacetic acid (tris-NTA). The SPR system consists of a programmable autosampler and a five-channel SPR instrument housing a 96-well microplate. Commercially available sensor chips were derived using Tris-NTA. They were then preloaded with Ni^2+^ by injecting NiCl_2_ solution. His-tagged S1 protein was immobilized in channel 2 (CH 2) by adding an S1 protein solution. His-tagged protein G was immobilized in CH 3 by flowing from the solution and His-tagged ACE2 protein was immobilized in CH 4 and CH 5. Anti-S1 antibody was injected into CH 2 and SPR signals were collected. Once the sample flows from CH 1 to 4, an antibody is detected in CH 2, antibody-neutralized viruses in CH 3, and free or partially neutralized viral particles in CH 4, whereas CH 5 is reserved for additional samples. The LOD was found to be 0.058 µg/mL for free anti-S1 antibody, whereas they were 126 TU/mL and 504 TU/mL for completely neutralized virus and free virus, respectively, in diluted serum samples.

Zhao et al. developed an LFIA assay for the detection of SARS-CoV-2 antibodies via colorimetric, photothermal, and SERS detection on gold-silver alloy hollow nanoshells (Au–Ag HNSs) [117]. Ag NPs were mixed with PVP and heated, and then chloroauric acid was added to form Au–Ag HNSs. Then, thiol-poly(ethylene glycol)-carboxyl (SH-PEG-COOH) were mixed and activated with EDC/NHS coupling. Finally, the protein was immobilized with the SARS-CoV-2 protein. The LFIA strips were fabricated using an NC membrane, an absorbent pad, a conjugate pad, and a sample pad. The T line was sprayed with ACE2 protein, whereas the S protein antibody was applied to the C line. The sample was added to the sample pad and changes in the T line were observed in 15 min. The neutralizing antibody competes with the immunoshells and prevents interaction with ACE2, resulting in no color formation on the T line. Photothermal detection was achieved by adjusting the shell-to-core ratio, while SERS detection was achieved by adding 4MBA to the nanoshell. The LOD was found to be 20 ng/mL in the linear range of 20–1500 ng/mL for SERS samples. Clinical samples from inactivated virus vaccinated and unvaccinated individuals were used, and no neutralizing antibody effect was observed in unvaccinated samples.

### Conclusion and future perspectives

COVID-19 has brought out a high demand for high-technology medical diagnostic devices, which also gave a hint for future pandemics that we need to be prepared. Due to severe deaths and it being highly contagious, early diagnosis platform has become very critical to prevent and control virus spread. RT-PCR has been widely used for SARS-CoV-2 virus from patients’ samples; however, it has bottle-neck that the equipment is very expensive and requires experienced personnel to operate in a laboratory environment. In addition, there was a stay-at-home order during the COVID-19 pandemic, emphasizing the need for low-cost, rapid, and point-of-care detection methods. Biosensors are great candidates for meeting the need for early diagnosis of diseases, with high selectivity and sensitivity especially when they are modified with novel nanomaterials. Biosensors have advantages over conventional methods in that they are accessible, affordable, and give fast results with the use of minimal sample volume.

This review highlighted the current technologies in optical biosensors and their concepts and detection ability for SARS-CoV-2 virus detection. Moreover, nanotechnology-based biosensors are discussed and their concepts regarding their modification steps, nanomaterial type, and biosensor characteristics are presented. The optical biosensors offer rapid detection at very low concentrations of the analytes. To achieve that, they require to be functionalized accordingly to absorb the target analyte. Most efforts have been given on biomarkers including antibodies, antigens, nucleic acids, and aptamers. The advantages and disadvantages of the biosensors are presented in Table 5.

**Table 5:** Advantages and disadvantages of biosensors

Biosensors	Advantages	Disadvantages
Antibody-based sensors	- High affinity for viruses and viral proteins - Specificity against analyte - Sensitive and fast detection [76]	- Detection of different epitopes in the same pathogen - Instability comparing with peptide-based probes [76]
Antigen-based sensors	- Provides good results in the long term as antigens are less likely to be degraded during transportation - Fast detection - Low cost [82]	- Shortage of test kits - Limited number of studies [82]
Nucleic acid–based sensors	- Fast analysis time - Low sample requirements [107] - Increased sensitivity due to self-amplification of aptamers - High specificity, affinity, and reproducibility - Ability to target large proteins thanks to its small molecular structure - Increased sensitivity and selectivity due to conformational change [119]	- Difficulty in detection due to mutation of viruses [81] - Difficulty in multi-target analysis [109] - Degradation by nucleases in vivo - Renal filtration and rapid clearance from the bloodstream limit sensor use - Prolonged aptamer production - Labor-intensive process [108]

Colorimetric methods and LFIAs provide advantages including miniaturization, portability, user-friendliness, and affordability for the analysis of body fluids such as nasal asperities to rapidly detect SARS-CoV-2 virus. On the other hand, a common challenge for optical biosensors is that measurements in complex samples are limited due to the sample preparation phase. Thus, the sample preparation step should be eliminated to facilitate its user-friendliness and simplicity in the future. Furthermore, aptamer-based nanobiosensors become popular due to their high affinity towards target analytes as low as femtomolar concentration in recent years. The critical point for the development of aptamer nanobiosensors is the proper selection of the aptamers. Application of aptamer-based biosensor is expected to increase due to their advantages over other affinity-based biosensors in the near future.

In recent years, artificial intelligence has exhibited great applications in various fields. Machine learning algorithms have the potential to predict many diseases; however, they are not sufficiently being used due to the poor database available online. With the use of a suitable machine learning model, monitoring of analyte levels in clinical samples of patients such as blood, urine, sweat, and exhaled breath could give information about infectious diseases at an early stage, providing the control of their spread and pandemics in the future. Also, selection of nanomaterial or biorecognition elements will enable for development of biosensors to diagnose COVID-19 owing to the use of artificial intelligence.

COVID-19’s machine learning can be categorized into four groups: forecasting, medical diagnosis, drug development, and contact tracing. The number of new infections is forecasted using recurrent neural networks and their variants. Studies show that the diagnosis of COVID-19 in chest X-rays can be performed with 99% accuracy thanks to deep learning software. By developing new chemical combinations with machine learning algorithms, it enables the development of new effective drugs at a lower cost and in less time. Finally, with digital proximity contact tracing, contact persons can be tracked with smart devices. Smart devices such as GPS, camera images, and cell phones are used for this [118].

Another promising technology to make significant progress in the fabrication of not only substrates of biosensors but also the transducer itself is 3/4D printing. In addition, it is possible to produce nanomaterials using 3D printing technology. 3D printing designs are helpful in creating hand-held rapid testing kits to assist the health care workers in the point-of-care diagnosis of COVID-19. With the advancements in nanostructures, smart manufacturing of biosensors will be achieved for the diagnosis of COVID-19 and other infectious diseases in the future.

## Data Availability

Availability of data and materials statement is not applicable.
